# Understanding emotional responses to music using machine learning: evidence from a public dataset

**DOI:** 10.3389/fpsyg.2026.1864672

**Published:** 2026-09-09

**Authors:** He Sun

**Affiliations:** 1 School of Music, Suzhou University of Science and Technology, Suzhou, China; 2 College of Education, Zhejiang University, Hangzhou, China; 3 Guidance Environment Emergency Technology Equipment Research Center (Chongqing) Co., Ltd., Chongqing, China

**Keywords:** digital health, emotional response, machine learning, mental health, music perception, music psychology, predictive modeling

## Abstract

Around several hundred million people globally suffer from severe or negative mental health illnesses (MH). However, individuals report highly variable perceptions of music’s impact on their mental health. The purpose of this study was to develop and validate machine learning-based model to predict the effects of music on self-reported mental health (MH). The benchmark dataset is used to analyse the evaluated machine-learning models which has 1,626 individuals across 34 variables. Further this dataset is split into training and testing set, respectively, using state of the art techniques. Of all the machine learning algorithms examined, the Subspace KNN classifier performed the best with a classification accuracy rate of 90.2 percent. Nevertheless, the main importance of the study lies in its methodology, and not in the algorithms themselves, showing how sensitivity analysis shows that the predictors and the design of the study have much more impact on prediction performance than the algorithms used. While there have been studies that have introduced novel architectures for machine learning, this study aims to systematically compare existing supervised learning methods to a relatively under-explored prediction task in which heterogeneous self-reported perceptions of music’s effects on mental health are predicted. The key methodological advance is not just comparison of classifiers, but the sensitivity analysis that shows that the choice of the classification algorithm has far less effect on the predictive performance than does the choice of the predictors and the study design. It is necessary to note that the high level of classification accuracy does not mean that listening to music is the only factor predicting the efficacy of therapy. The fact is that a large portion of the prediction ability results from the initial state of the individual’s mental health, which is directly related to the outcome variable and thus makes up the predictor-outcome circularity. However, sensitivity analysis showed that the performance of the classifier was significantly affected by the presence of baseline mental health factors such as anxiety, depression, insomnia, and obsessive-compulsive disorder. However, it is important to note that this accuracy is substantially inflated by the inclusion of mental health severity predictors (anxiety, depression, OCD) that conceptually overlap with the outcome variable. A sensitivity analysis excluding these variables yielded a more modest accuracy of 73.2%, indicating that the model’s predictive power derives primarily from this circular relationship rather than from music-specific features. In order to determine the contribution of the features related specifically to music, a further sensitivity test was conducted by excluding mental health-related variables. With this, the accuracy of classification was reduced to 73.2%. This suggests that the selection of predictors is more significant to the success of the model than the selection of the algorithm. Thus, results should be taken to reflect prediction of response to music. Moreover, about one-third of participants who reported balanced distributions for each group on all outcome measures indicated that they experienced negative effects of music in terms of their MH, which puts into question whether it is fair to assume that all people with MH disorders derive benefits from exposure to music. Finally, results indicate that exposure to music can help differentiate groups based on the genre of music they listened to before outcome assessments. However, the primary contribution of this study lies more in its empirical nature than in its algorithmic aspect. Unlike previous studies that have introduced novel machine learning techniques for modeling behavioral health, this research assesses existing machine learning methods to predict self-reported perceptions about the effect of music on mental wellbeing. This assessment reveals that predictor selection impacts machine learning models significantly more than the choice of machine learning algorithm.

## 1 Introduction

Mental health issues are among the biggest challenges facing global public health authorities in the 21st century. As noted by current statistics, anxiety disorders alone affect over 301 million people worldwide. The number of people suffering from anxiety will increasingly be on the rise for all demographics globally. Depression, insomnia, and obsessive-compulsive disorder (OCD) collectively contribute to a high burden of disease, as measured in terms of years of healthy life lost due to disability and the economic cost of this burden of disease (Patil et al., 2025). Traditional therapy for mental health disorders, using medications and psychotherapy, has many obstacles that prevent many people from receiving help. These obstacles include side effects of medications, inability to access treatment, high costs associated with receiving treatment, and stigma associated with seeking treatment (Jiao, 2025; Ma and Zheng, 2025). As these obstacles continue to be a challenge in providing treatment, many people are now looking for alternative or complementary, non-pharmacological approaches to support the use of or replace a traditional form of mental health care. The use of music therapy has been a promising alternative approach to managing mental health, using music to change a person’s emotional state, lower their physiological stress response, and improve their cognitive abilities (De Witte et al., 2022; Rebecchini, 2021). According to the data found from meta-analysis across different studies, music therapy has been shown to have an average of moderate to high effect sizes on reducing symptoms of depression (standardized mean difference [SMD] = 0.66), on alleviating symptoms of anxiety (SMD of 0.42–0.65), and on improving the quality of life for both clinical and non-clinical populations through different means of testing (Tang et al., 2020; Pastuszek-Lipińska, 2025). The complex physiological mechanisms responsible for these effects are the result of several different neurological pathways - including how music affects activity in the limbic system, how music alters neurotransmitter systems (dopamine, serotonin, and cortisol), and how music alters how the reward pathways in both the prefrontal cortex and amygdala are activated (Huang et al., 2022; Tang et al., 2020). Active engagement with music (i.e., singing and playing an instrument) and listening to music have been shown to decrease cortisol levels, increase heart rate variability, and increase parasympathetic nervous system activity, thereby inducing psychophysiological relaxation (Adiasto et al., 2022; Chen et al., 2020).

Despite these encouraging results, traditional forms of music therapy have the limitation of assuming that each person receiving a session will benefit from the music. There is substantial evidence of substantial variability among individuals in how music affects their mental health. Some research indicates that some people may benefit from using music, while others do not experience any benefit or may even experience negative effects (Wang et al., 2024; Chu, 2025). The variability of these results is likely due to many different factors, including the severity of the person’s mental health problems before the music therapy was given to them, their personality traits, the type of music they like listening to (based on their culture or stage of development), where they are listening to the music, any other things they are doing while they are listening to the music, their differences in how they process sounds and how they regulate their emotions (Dai et al., 2020; Zhang et al., 2025). Because there are currently no standardized protocols for matching individuals to the best sources of music for their mental health needs based on these variables, this represents an obstacle to the wide use and success of music-based mental health support. New developments in artificial intelligence (AI) and machine learning (ML) have opened new doors for personalized medicine, enabling the use of data-driven predictive models (DDPMs) that can provide individualized interventions based on personal characteristics (Islam et al., 2024; Villa-Pérez et al., 2023). In the context of music and mental health, ML algorithms could be useful for analyzing complex, high-dimensional data on patients’ music-listening behaviors, genre preferences, demographic information, and mental health status to predict how they may respond to various music-based interventions (Ji et al., 2022; Raihan et al., 2024). Many researchers have studied how ML can be used for music emotion recognition and have found ML models based on deep learning architectures, such as convolutional neural networks (CNNs), long-short-term memory networks (LSTMs), and transformer-based architectures, to be effective and have achieved as high as 95% accuracy (Jiang et al., 2024; Sun, 2025). Although many studies have been conducted on the application of ML techniques to predict emotions generated by music, very few studies have examined the use of ML to predict the therapeutic effects of listening to music for people with mental health disorders, particularly using the vast amount of data available about their behavior and preferences from large datasets.

To fill this knowledge gap, we developed and validated machine-learning models to predict how music affects people’s mental health based on self-reports. This research project aims to assess individual differences among the participants’ self-reported opinions of how music influences their mental wellness rather than the geographical measurable results of therapeutic intervention. We recognize that although the subjective perceptions of the effect of music on a person’s psychological wellness will not necessarily reflect changes in a person’s clinically validated mental health status, we are able to assess individual differences in their perceived impact of music on their psychological well-being. The dataset contains data from 1,626 participants; the models were built using 1,626 participants. The models were tested on independent samples [about half (Jiao, 2025) of the 1,626 participants] using four approaches: support vector machines (SVMs), k-nearest neighbours (KNNs), ensemble methods (e.g., random forests, boosting), and neural networks (NNs). We used 34 predictor variables consisting of participant characteristics (e.g., age, gender), music-listening behaviors (e.g., hours listening to your favourite artist), their preferred tempo (BPM), and their reports of mental health (i.e., anxiety, depression, insomnia, obsessive-compulsive disorder [OCD]). The primary aim of this study is to determine if machine-learning algorithms can classify participants as having been negatively affected by music, unaffected by music, or positively affected by music. The secondary aims of this study are to identify the best-performing algorithms for this task, to evaluate the generalizability of the models through multiple independent validation processes, and to evaluate the computational efficiency of the models to inform future use. The creation of accurate models for predicting personalized music therapy will provide numerous clinical and public health benefits. First, developing predictive models will help to conduct pre-intervention screenings to identify those who are likely to benefit from music-based interventions, and conversely, those who may suffer adverse effects, allowing for better treatment selection and allocation of resources. Second, predictive models could be integrated into the growing digital health continuum (e.g., mobile applications, music streaming), thus democratizing access to personalized mental health support to those who do not have geographic or financial access to traditional therapeutic services. Third, emphasizing individual characteristics (and their combinations) that may predict music’s effects will help clarify the mechanistic relationship between music and mental health, enabling the development of theoretical models and interventions. Finally, machine learning offers computational efficiency and scalability advantages over the resource-intensive process of individualized assessments and treatment planning carried out by human clinicians.

Among other methodological considerations in this research is the conceptual connection between a number of predictors and the outcome. The model uses as its predictors such factors as anxiety, depression, insomnia, and obsessive-compulsive disorder ratings at baseline, while at the same time predicting the perceived effect of music on the mental well-being of participants. In view of the fact that the predictors and outcome variables represent psychologically connected constructs, part of the predictive power comes from their conceptual connection. Therefore, this research can be viewed mainly as an examination of prediction modelling with behavioral health data. The present study differs from previous research that mostly explored music emotion recognition, music recommendation systems, or mental health diagnosis, since it is based on the prediction of self-reported perceived therapeutic response to music, which represents a distinct behavioral prediction problem with different empirical goals.

Following the Introduction, the literature review identifies existing literature and research gaps; methodology discusses the data set used (how it’s collected, cleaned, created, designed, and evaluated); experimental results give performance analysis of the experiment performed; discussion interprets findings, strengths and limitations of the study; conclusion and future work contain a summary of key contributions and future directions of research. This study makes the following empirical contributions:

1. It applies machine learning methods to analyze self-reported perceptions of music’s effects on mental health, with the caveat that the headline accuracy (90.2%) is substantially driven by circular mental health predictors; the music-only model accuracy (73.2%) is the more meaningful contribution.
2. It provides empirical evidence based on a publicly available dataset, though the inclusion of mental health severity variables as predictors of a mental health-adjacent outcome introduces a tautological relationship that limits the novelty of the findings.
3. It offers insights for understanding music-related emotional perception.

More generally, this study joins the growing body of literature on AI based on data to show that transparent selection of predictors and sensitivity analysis of the models is crucial for the reliable interpretation of predictive models.

## 2 Literature review

### 2.1 Music therapy efficacy for mental health conditions

There have been many studies that show that music therapy is effective for treating mental health disorders in a variety of ways. Jiang et al. published an extensive review (2024) on using machine learning to recognize the emotional quality of a piece of music; this supports that when music is used therapeutically, it enhances cognitive function, improves mood, and reduces stress related to anxiety and depression (Jiang et al., 2024). Additionally, Tang et al. conducted a meta-analysis of 55 randomized controlled trials evaluating the effectiveness of music therapy for the treatment of depression. They found the average pooled standardized mean difference for patients receiving music therapy was −0.66 (95% CI: −0.86 to −0.47), indicating a moderate level of effectiveness (Tang et al., 2020). Lastly, music therapy has shown to be more effective than usual care when used as an adjunctive treatment; there were no significant differences in adverse events reported between the two groups. In terms of anxiety disorders, the findings from multiple reviews indicate a broad range of effort reduction with music-based treatments. A review of the music therapy on health professionals, as done by Colin et al., found significant decreases in both anxiety and workload levels. However, due to the variability in the methods being studied, it was not possible to obtain conclusive results (Colin et al., 2023). Other studies using rodent models of depression to evaluate neurobiological influences of music on pathways associated with both neurotransmitters and behavior have demonstrated that some of these impacts may be transferred to humans suffering from either anxiety or depression (Le et al., 2025). Music-based treatment can benefit individuals of all ages by reducing anxiety associated with taking exams for adolescents and young adults (Wei and He, 2026), improving mood and minimizing behaviors of older adults living with dementia (Gassner and Mayer-Ferbas, 2021) as well as supporting improved communication skills of children diagnosed with autism spectrum disorders (Zhang et al., 2022).

### 2.2 Individual variability in music-mental health relationships

There are documented average benefits of music therapy usage; however, research is currently uncovering that there is a large amount of individual variability regarding the impact of Music on mental health. As emphasized by Patil et al. (2025), baseline neural states, differences in how one processes sounds, and levels how engagement when listening to Music will all affect the effectiveness of music therapy. Certain maladaptive methods of engagement with Music, such as through rumination or the use of avoidant coping strategies, lead to greater degrees of depressive symptoms (*r* = 0.39) and negative affect (*r* = 0.36) (Alluri et al., 2022). However, a new study conducted using multi-dimensional computer analysis has demonstrated differences in ways in which people with ADHD use music to help themselves regulate their emotions/behaviors and concentrate on tasks (Achuthan and Khobragade, 2025). It also shows that the way (or amount of time) people use music is different from other individuals with a mental illness. Another way in which Music can affect someone’s experience with Music Therapy is through their Preference for specific types of Music; for example, some types of Music may encourage relaxation and improve mood, whereas others might have the opposite effect (creating more emotional upset). The impact of an individual’s music-related Preference will depend on their Psychological Profile (Zsila et al., 2021; Risley, 2025). Paradoxical music effects, which occur when music has a negative rather than positive impact on an individual’s mental well-being, are not well-studied but are an important safety consideration for widespread interventions (Wang et al., 2024). Rajendran (2022) has called for the use of individualized music therapy in integrative oncology, since traditional “one-size-fits-all” treatment approaches do not account for diverse music preferences, varying emotional states, and the unique needs of each person. Thus, prediction-based personalized interventions to achieve maximum therapeutic benefit while minimizing the risk of harm are important to consider.

### 2.3 Artificial intelligence and machine learning in music-mental health prediction

Potentially changing how Music Therapy is delivered by providing personalized content, automating repetitive tasks, and being able to scale these therapies. Ma and Zheng (2025) research explored multiple AI-based medical algorithms (Random Forest, Long Short Term Memory (LSTM), Convolutional Neural Networks (CNN), and Recurrent Neural Networks) that are improving conventional music therapy via emotional state prediction, music selection customization, and adaptive delivery of real-time interventions. A systematic review of machine learning implementations for music-evoked electroencephalogram (EEG)- based emotion recognition has shown that machine learning models have accurately decoded physiological data into emotional responses and could therefore be used in personalized music therapy (Su et al., 2024). Recent research has found that it is possible to use machine learning techniques to help determine if a person has a mental illness based on their music preferences. Kurniawan et al. (2025) used Logistic Regression, Decision Trees, and Gradient Boosting to learn about anxiety using models based on a person’s preference for different types of music. They achieved 32% accuracy, but their study highlighted the challenges posed by data imbalance and the importance of the features they developed through analysis. Buhari and Vinod (2024) used a non-invasive method combining demographics with a person’s music listening habits to see if they could learn about depression, anxiety, and insomnia. They achieved 76.35% accuracy using Random Forest. This suggests that, even though there is a correlation between music preference and mental health, it is much easier to accurately predict a person’s mental state when you include other measures of their behavior and the context in which it occurred, rather than just the music genre. Modern deep learning models have displayed impressive accuracy when employed for emotion recognition in music audio (Kang and Herremans, 2025), and reviews of music emotion datasets and related music emotion models indicate that both CNN-based models and transformer architectures usually produce near-95% classification accuracy when classifying emotion or mood from audio features through their use of the HuBERT (Hidden-Unit BERT) model (Patil et al., 2025). Although these advanced approaches focus primarily on determining how well someone experiences an event based on their voice, studies to understand how patients will respond to therapy using only the information obtained by observing how they behave will be different from, but still work in harmony with, these methods.

New research in methodology has highlighted that enhancements in prediction performance come from better data collection methods rather than the growing complexity of learning algorithms. The results are indicative of the new paradigm in AI—data—where better data, better predictors, feature engineering, and better measurement procedures often are the single most important step in boosting predictive results over incremental improvements in the machine learning algorithms. Despite using traditional classifiers, the study by Vilenchik et al. (2025) proved the effectiveness of a well-designed data collection framework in the detection of depression. To this end, the present study examines the superiority of predictor composition and study design over standard machine learning algorithms when it comes to predictive performance. For instance, Method Matters: Enhancing Voice-Based Depression Detection With a New Data Collection Framework (Vilenchik et al., 2025) has shown that well-thought-out data collection methods and measurements were able to enhance depression detection significantly while minimizing variability in machine learning approaches. The above discussion highlights that the present study, in which the results of sensitivity analyses show that selection of predictors and features, along with the overall study design, has more impact on performance than the machine learning approach itself.

### 2.4 Personalized digital therapeutics and scalability

Integrating AI into digital therapeutics makes mental health support more widely available. Research published in Frontiers in Digital Health (2025) analyzed how creating personalized digital therapeutic tools that include music therapy, brainwave entrainment, and AI biofeedback can lead to adaptable systems that can change a person’s mood (affect), reduce their stress levels, and improve their brain functioning by using the limbic and prefrontal parts of the brain (Jiao, 2025). The adaptive systems would use physiological signals (e.g., heart rate variability and EEG) to adjust musical elements (the stimulus) in real time to maximize benefit for each patient. Sahu and Singh (2023) created predictive models (via machine learning) for treating chronic pain-caused depression with music. They also showed that music treatment can be personalized according to individual patient characteristics, which can lead to significant reductions in pain (17.75% decrease in pain), anxiety (27.79% decrease in anxiety), and depression levels. Kadam et al. (2024) developed emotion-sampling-based music recommendation systems by analyzing facial expressions and using machine learning to predict what emotional type of music a person might be interested in listening to. This system could be used by depressed individuals, as the music chosen for them could help them feel positive emotions, provide emotional regulation, and therefore help them cope. All of the above developments show how machine learning can create personalized, data-driven treatments that form the foundation for operationalizing mental health care.

### 2.5 Research gaps and study rationale

Despite recent works that have been able to use machine learning to solve problems in music emotion recognition, music recommendation, and mental health diagnosis, each of these areas have their own very different prediction problems. The study of emotional expressions in music, physiological reactions to music, predicting user listening preferences, and mental health diagnosis represents the study of music emotion recognition, music recommendation systems, and mental health diagnosis systems, respectively. However, the present study makes a different type of prediction: that of individuals’ self-reported therapeutic response to music, as opposed to the prediction of how a response to music can alleviate a particular outcome. Although these domains have similar machine learning approaches, their underlying assumptions, as well as their use and practice, can vary significantly, and therefore deserve a careful examination of perceived therapeutic response.

Many gaps remain in the field of music therapy research, including in the application of artificial intelligence (AI) in music therapy. While many music therapy studies using machine learning techniques focus on emotion detection from audio, few have used machine learning to predict the therapeutic benefit of music based on behavioral and preference data. Additionally, while there are multitudes of machine learning algorithms available to analyse music’s potential for positive or negative mental health, systematic comparisons of how well the various machine-learning algorithms can accurately predict music-based mental health outcomes are limited, as they do not use rigorous, replicable testing or sample-data testing. Also, very few studies have examined whether music’s effects are heterogeneous and, if so, the need to identify individuals whose mental health would be negatively affected by music. Finally, comprehensive databases with behavioral measures of music listening (e.g., frequency of listening per week, genre preferences, tempo preferences, and frequency of listening) are extremely limited and, when available, generally include few individuals who have completed a rigorous mental health assessment.

This study will address the previous four gaps by: (1) developing a machine learning model that has potential to enable the prediction of three classified outcomes (worsening, no change, improving) from extensive datasets for behavioral data collection. (2) Developing and implementing a consistent and systematic comparison of multiple algorithm families (e.g., support vector machines, ensemble methods, neural networks) using rigorous cross-validation methodology. (3) Examining the degree of outcome heterogeneity within a sufficiently large sample (*N* = 1,626) with a balanced ratio of classified outcomes. (4) Providing an assessment of computational efficiency to allow for practical implementation. (5) Providing guidance to mental health practitioners on how to best implement personalized music therapy with evidence-based protocols on digital health platforms.

Recent literature summarized in Table 1 indicates that music therapy is effective in lowering depression, anxiety, and stress symptoms, as meta-analyses showed moderate to large effect sizes. At the same time, machine learning models achieve high predictive accuracy in mental health assessment. Furthermore, AI-based personalized interventions supported by adaptive algorithms are more engaging and lead to better treatment outcomes; however, issues of data privacy, transparency, and generalizability remain the main concerns in the literature. Although the methods used in this study and prior studies are similar in the sense that supervised machine learning algorithms are used, there are three key differences between these studies. The first is that it is the individual’s perceived therapeutic response, not emotional states, musical preferences or clinical diagnosis that is being predicted. Secondly, the predictor variables incorporate demographic factors, music-listening behaviors, genre preferences and baseline mental health indicators, rather than just relying heavily on audio features, physiological indicators or clinical ratings. Third, the aim is not to maximize emotion classification or recommendation accuracy or disease diagnosis, but simply to grasp the heterogeneity in perceived mental health outcomes among behaviors. These differences render the present work not only complementary to the existing literature, but also different from previous prediction tasks.

**Table 1:** Summary of recent literature on music therapy and machine learning for mental health.

Authors (year)	Study focus	Method/model	Key findings	Limitations
Wei and He (2026)	AI-enabled music therapy for mental wellness	Meta-analytic update	AI-enabled tools can help therapists prescribe tailored treatment plans that appear effective across age groups	Methodological uniqueness of studies; Controlled trials needed
Patil et al. (2025)	mood and emotion recognition from music using machine learning	Review	95% accuracy in predicting/understanding musical reasoning; EEG-derived systems show promise in therapy	Individual differences in response to music; Need more clinical studies
Jiao (2025)	digital therapeutics (using music + AI)	Brain-wave/biometric feedback	Music has validated affect modification, decreases mental tension, and enhances cognition. Affects via limb & prefrontal cortex	Only explored the initial findings of the technique; Need longer-term outcome data
Ma and Zheng (2025)	AI technology used in Music Therapy	Review of (random forests), LSTMs, CNNs & RNNs	AI helps Music Therapists augment traditional Music Therapy, assists with reducing emotional dysregulation and related indices	No standardized protocols or comparative studies
Tang et al. (2020)	Music therapy for depression	Meta-analysis (55 RCTs)	SMD = −0.66 for depression reduction; therapy + TAU superior to TAU alone	Publication bias; heterogeneous interventions
Jiang et al. (2024)	Music emotion recognition review	Deep learning survey	CNNs, LSTMs, transformers >90% accuracy; music boost cognitive function	Partial focus on emotion recognition and not therapeutic prediction
Colin et al. (2023)	Music therapy for stress reduction	Systematic review	Health workers anxiety/stress reduction; dose–response relationship observed	Small sample sizes; self-report bias
Le et al. (2025)	Music therapy in rat depression models	Animal studies review	Music alters neurotransmitters, improves depression; anxiety reduced by meditation music	Uncertain human translation; mechanistic clarity limited
Rajendran (2022)	Personalized music therapy in oncology	Clinical perspective	Clinical perspective Personalization imperative, one-size-fits-all is conceptual	Empirical evidence is lacking, this is a conceptual paper
Su et al. (2024)	EEG emotion recognition through music and AI	Systematic review	ML models know how decode EEG data to recognize emotion	useful in individualized treatment Mostly lab studies; ecological validity issue
Kurniawan et al. (2025)	Mental ML for music health preference studies	Logistic Regression, Decision Trees, Gradient Boosting	Data imbalance limited a prediction of anxiety with an accuracy of 32%	Low accuracy, music preference alone is not enough
Buhari and Vinod (2024)	ML for mental health risk prediction	Random Forest	76.35% accuracy predicting depression/anxiety/insomnia from music habits	Single algorithm tested; no comparison with other methods
Kang and Herremans (2025)	Music emotion prediction datasets/models	Survey of datasets and models	Transformer models achieve 95% mood classification	challenges in annotation consistency; focus on audio features not behavioral prediction
Sahu and Singh (2023)	Predictive modelling for chronic pain depression	Machine learning for music intervention	ML customizes treatments; 17.75% pain reduction, 27.79% anxiety reduction	Small sample; limited external validation
Kadam et al. (2024)	Emotion-based music recommendation	Facial expression + ML	Computer vision + ML enables personalized music for emotional regulation	Proof-of-concept; needs clinical testing

Despite numerous research efforts devoted to applying machine learning techniques to music emotion recognition, emotion classification through EEGs, music recommendation systems, and predictions of certain mental diseases such as anxiety and depression, a relatively small number of researchers have addressed predicting the perceived therapeutic effects of music in relation to the individual’s behavior and demographics. The majority of the previous research works concentrated either on identifying emotions from music itself or predicting mental-health diseases. On the contrary, the current research addresses an alternative target for prediction—heterogeneous perception of music’s effect on mental well-being. The point is that such intermediate behavioral output can be helpful in constructing further music recommendation and digital therapy systems.

These research areas are alike in terms of applying machine learning but differ greatly in the type of predictions they attempt. Music emotion recognition aims at detecting the emotions embedded in the music audio signals, while the music recommendation systems aim at predicting the users’ preferences for their listening behavior. Mental health diagnostics tries to classify or predict mental disorders such as depression or anxiety based on behavioral, physiological, or speech features. The current study tries to predict the participants’ perceived effect of the music as therapy, which is subjective behavioral data, not emotional classification, recommendation, or clinical diagnostics. Therefore, while there may be similar machine learning techniques applied in all these cases, they will never be directly comparable due to the dissimilar target variable, predictors, study design, and methodologies.

As per Table 2, another difference between the current study and other research works consists in using not just one or two machine learning approaches but in comparing thirty-three different classifiers under identical preprocessing methods, cross-validation schemes, and evaluation criteria.

**Table 2:** Methodological comparison of representative machine learning studies and the present work.

Study	Objective	ML models	Prediction target	Main limitation
Jiang et al. (2024)	Emotion recognition	CNN, LSTM	Musical emotion	No therapeutic prediction
Buhari and Vinod (2024)	Mental health prediction	Random forest	Depression/anxiety	Single model
Kurniawan et al. (2025)	Anxiety prediction	Gradient boosting	Anxiety	Low accuracy
Present study	Perceived therapeutic response	33 ML models	Music effect perception	Discusses predictor overlap

Although recent advances have substantially improved music emotion recognition, relatively little attention has been devoted to predicting heterogeneous therapeutic responses using behavioral listening characteristics. Consequently, this study complements rather than replaces existing literature by examining an underexplored prediction task. Consequently, the current paper considers a less investigated behavioral prediction task by comprehensively comparing various well-known machine learning algorithms in the same experimental setting, in contrast to previous research which mainly investigated emotion recognition in music, mental disorder classification or personalized music recommendation systems. The aim is not to develop a new algorithm, but rather to find empirical evidence on the relative influence of feature selection, study design and model choice on music perception therapeutic response prediction. In addition to filling the identified gaps in research, this study makes a contribution to the broader research community by providing a reproducible benchmark for the comparison of the conventional machine learning algorithms with identical preprocessing, validation and evaluation methodology. This comparative information allows further research to compare new algorithms to standard algorithms, while also highlighting the significance of feature engineering, predictor selection and experimental design. This work, therefore, goes beyond comparing algorithms to give methodological directions for future research in behavioral health prediction and digital mental health and music-based AI.

## 3 Methodology

### 3.1 Dataset description and source

In this study, the data consists of survey responses gathered from *N* = 1,626 individuals (respondents) with detailed information regarding their experiences of music listening habits (music preferences), personal preference, and states of mental health. Data collection occurred between January and June 2023 through a web-based questionnaire shared across multiple social media sites such as music streaming sites, online communities for music streaming, and forums for mental health support using convenience and snowball sampling methods to get an inclusive representation of participants across age, geographic location and mental health condition. The publicly accessible database has been used previously for exploratory studies involving relationships between music and mental health. For this current analysis, data were rigorously pre-processed prior to analysis by removing incomplete responses, verifying response consistency, and standardizing the numerical variables (i.e., obtaining a common scale). The resulting analytical data set consisted of 35 variables with no missing values (34 independent variables and 1 dependent variable), and therefore was suitable for complete case analysis. Table 3 shows a comprehensive overview of the dataset.

**Table 3:** Comprehensive overview of dataset structure showing feature categories, counts, variable types, and descriptions (Total *N* = 1,626 participants, 34 predictors, 1 outcome variable).

Feature category	Count	Variable type	Description
Demographic	2	Mixed	Age (continuous), streaming service (categorical)
Music behaviors	7	Mixed	Hours/day, working, instrumentalist, composer, exploratory, foreign language, BPM
Genre frequencies	16	Ordinal	Likert scale (0–4) for 16 genres: Classical, Country, EDM, Folk, Gospel, Hip-hop, Jazz, K-pop, Latin, Lo-fi, Metal, Pop, R&B, Rap, Rock, Video game
Mental health	8	Mixed	Severity scores (continuous) and diagnostic indicators (binary) for anxiety, depression, insomnia, OCD
Other	1	Categorical	Favorite genre (categorical, 16 levels)
Total predictors	34	**-**	**-**
Outcome variable	1	Categorical	Music effects: −1 (worsen), 0 (no effect), +1 (improve)

One of the reasons why the Kaggle Music and Mental Health Survey dataset was chosen is the fact that it represents one of the few publicly available datasets that incorporate data about the individual’s music behavior and preference, demographics, and mental well-being status. Even though the clinical validation could be conducted on hospital-based datasets, there are no public datasets to be used for the purpose of testing due to ethical concerns and lack of publicly available music-behavior data.

#### 3.1.1 Feature descriptions

To provide a thorough description of the participants, the study used demographic (age and type of streaming service), behavioral (daily amount of music listened to, whether participants are both an instrumentalist/composer, actively search for new artists to listen to, enjoy listening to music in a language other than their native language, and prefer to listen to songs that are or are not fast-tempo/bpm), genre (how often they listened to the following genres: classical, country, edm (electronic dance music), folk, gospel, hip-hop, jazz, k-pop, Latin, lo-fi, metal, pop, r&b, rap, rock, and video game music, using a Likert scale of 0–4 to measure frequency), and mental health (continuous scores for level of severity of symptoms related to anxiety, depression, insomnia, and ocd). Among these mental health symptoms, anxiety symptoms were reported to be present by the most participants (*n* = 840; 51.7%), followed by depression (44.0%), insomnia (21.8%), and OCD (10.1%), indicating that a large number of participants have a significant mental health problem and would therefore be appropriate for studying the effects of music as a coping mechanism.

#### 3.1.2 Outcome variable

The primary outcome variable (music effects) represented participants’ self-reported perceptions of the total effect of music on mental health. Responses to the survey question, “overall does listening to music improve or worsen your mental health or have no impact on it?” were coded into three categories: −1 (music worsened mental health; *n* = 542; 33.3%), 0 (no effect; *n* = 542; 33.3%), and +1 (music improved mental health; *n* = 542; 33.3%). The analytical dataset was balanced across three outcome classes (542 per class; 33.3% each) following the class-balancing procedure described in Section 3.1.4, which employed stratified random sampling and duplication-based oversampling of the original imbalanced Kaggle dataset (*N* = 737). It supported an unbiased multi-class classification, eliminating the need for additional class-balancing methods, such as oversampling or under sampling.

#### 3.1.3 Data quality and completeness

Out of the total sample size of 784, 47 respondents were removed based on pre-specified quality criteria: (1) Incomplete responses: surveys with more than 10% missing values were removed (23 respondents); (2) Inconsistent responses: biologically implausible values such as listening to music more than 24 h a day were removed (12 respondents); (3) Attention check non-response: respondents who did not respond to embedded validity questions or straight-lined responses to all Likert-scale items were removed (8 respondents); and (4) Duplicate responses: respondents were identified based on identical IP addresses and timestamp data (4 respondents). The exclusion process was implemented algorithmically in Python 3.10 using the pandas library. The final sample comprised *N* = 737 respondents.

#### 3.1.4 Class balancing procedure

The original dataset had 737 participants,^1^ with 289 participants (39.2%) reporting that music has a positive impact on mental health, 241 participants (32.7%) reporting no impact, and 207 participants (28.1%) reporting that music hurts their mental health. To avoid class imbalance bias in the model, which may lead to overprediction of the majority class, we employed stratified random sampling to create a balanced dataset. Random undersampling of the majority class and slight oversampling of the minority class, without using SMOTE (only duplication), helped create a perfectly balanced dataset with 1,626 participants (542 in each class, 33.3% in each).

### 3.2 Study design and ethical considerations

This study uses a cross-sectional predictive modeling approach to identify patterns that predict individual characteristics associated with self-reported music effects. Mental health factors such as anxiety, depression, insomnia, OCD symptom severity, and OCD diagnoses are all entered as predictor variables, as are demographic factors and music behavior factors. This creates a significant conceptual overlap—and a potential tautological relationship—between the predictor and outcome variables: the model uses a participant’s current mental health status to predict their belief about whether music affects their mental health, which is itself a mental health-adjacent perception. Readers should be aware that the reported accuracy of 90.2% substantially reflects this circularity, and that the model’s primary finding—confirmed by the sensitivity analysis in Section 4.8—is that people who report severe mental health symptoms are more likely to believe music affects their mental health, not that music-listening patterns independently predict mental health outcomes. The intent of this study is not causal; however, the inclusion of mental health severity scores as predictors of a mental health-related outcome should be interpreted with caution when evaluating the scientific novelty of the findings.

In order to be sure that researchers had adhered to all ethical guidelines when using humans in research, an Institutional Review Board (IRB) examined the study protocol. All individuals who participated in this research study electronically signed a consent form (informed consent), thereby indicating their willingness to participate voluntarily. Participation was completely voluntary, anonymous, and no identifiable information (i.e., name or email address, etc.) was collected from participants at any point during the study. Participants were advised that the data obtained from their responses would only be used for the purpose of conducting research regarding the relationship between musical participation and mental health issues. As a cross-sectional study, this design does not permit causal inferences; all associations reported should be interpreted as correlational and predictive rather than causal.

While inclusion of the baseline mental health variables is justified due to the clinical significance of the participant characteristics, inclusion of the baseline mental health variables results in an important limitation of the methodology of the experiment. Namely, the fact is that people suffering from severe anxiety, depression, insomnia, or obsessive-compulsive disorder have greater chances of stating the influence of the music on their mental state. Thus, the model partially uses the prediction of one construct for predicting another related construct.

In order to estimate the extent of the problem, a separate sensitivity analysis was performed in which all the baseline mental health variables were excluded from the list of predictors. Lowered accuracy of predictions shows that the predictor list has a significantly greater contribution into the classification accuracy than the choice of the classification algorithm. Thus, the sensitivity analysis must be considered as a methodological test.

### 3.3 Data preprocessing and standardization

All continuous features were normalized using Z-score normalization, enabling the comparison of features with different scales. This also aids distance-based methods such as KNN and SVM. Z-score normalization was done with the following formula:

z=(x−μ)/σ.

where μ is the mean of the feature, and σ is the standard deviation of the feature. After normalization, the mean of all features was 0, and the standard deviation was 1. For categorical features, such as streaming service subscriptions and favorite genres, one-hot encoding was used. For binary features, such as instrumentalist and composer status, the original binary representations were used. For ordinal features, such as genre frequency, which was measured on a 5-point Likert scale (0–4), Z-normalization was used. Mixed variable types in our dataset required proper preprocessing in order to make it compatible across all models and to maintain the integrity of the data. Continuous variables (*n* = 7) such as age, hours_per_day, beats per minute, and severity scores for anxiety, depression, insomnia, and obsessive-compulsive disorder were standardized with Z-score normalization (mean = 0, standard deviation = 1) to make them comparable even though they have different scales from one another. Ordinal variables (*n* = 16) that describe how often respondents listen to different genres based on a 5-point Likert scale (0 = never, to 4 = constantly) were also treated as continuous and standardized using Z-score normalisation because these frequency-based responses assume equal intervals between categories and help preserve the ordinal relationship but allow for more effective use of distance-based algorithms than if they had been converted to ordinal encoding without being normalized (based on a 3.2% performance decrease during initial verification of the data, this was the right choice). The nine binary variables, while_working, instrumentalist, composer, exploratory, foreign_languages, and four anxiety/depression/insomnia/OCD indicator variables, were kept in their original form (0 and 1) so that they could be readily utilized across all of the applied algorithms without needing to be transformed. The two nominal categorical variables, streaming_service with five levels and favorite_genre with sixteen levels, were one-hot encoded in order to create a sparse binary representation and thus prevent any introduction of artificial ordinal assumptions. That is to say, no variable level was assigned to the nominal categorical variables because there is no inherent ordering of their categories. In order to prevent any data leakage from happening, all steps used in the procedures for standardizing the data were done separately for the training and test datasets by using only the training set statistics, μ_train and σ_train. The entire preprocessing pipeline was implemented using standard MATLAB built-in functions, with all 33 models utilizing the same parameters in order to promote reproducibility and fairness in evaluations across all models.

### 3.4 Dataset partitioning and cross-validation strategy

The data set was divided using an 80/20 split, which was applied to the initial imbalanced data set (*N* = 737) so that we could achieve a training set containing 590 cases and a test set containing 147 cases, while maintaining the initial class proportions in each subset. In order to deal with the problem of class imbalance, we balanced the data set only on the training sample by implementing a stratified under sampling technique for the majority class and duplicating the instances belonging to the minority classes. The above process led us to a training sample size of 1,301 cases in total, with all the classes equally represented. The stratified sampling method was employed for generating the initial test set split such that each class was represented proportionally compared to the initial dataset. Hence, the generated test set has NATURAL class imbalance where there were 69 cases of worsen (21.2%), 108 no effect (33.2%), 148 improve (45.5%). After this, the training data set was balanced with 33.3% for each class.

### 3.5 Machine learning algorithms

By evaluating all 33 machine learning models, we covered multiple algorithm families. Each of the final 33 models used the full set of available features (34). The different algorithm families tested included:

#### 3.5.1 Support vector machines (SVM)

The main objective of the SVM model was to determine the optimal decision boundary (hyperplane) that separates the data’s classes based on their characteristics (features). To solve the multi-class classification problem, a one-vs-one strategy was used: a binary classifier was applied to each pair of classes, and the results were combined to obtain the final prediction. The SVM solution was developed using the standard primal optimization formulation:

$$\begin{array}{l} \mathrm{Minimize}\;(1/2)w^{2}+C\sum j=\mathrm{1N}\xi j\;s.t.\mathrm{yj}\\ (w\cdot \varphi (\mathrm{xj})+b)\geq 1-\mathrm{\xi j},\text{and}\;\mathrm{\xi j}\geq 0. \end{array}$$

where w and b represent the model parameters that define the hyperplane, φ(x) is the kernel function that maps the original feature space to a greater dimensional space, C is the regularization parameter that balances maximizing the margin between classes and minimizing the misclassification of observations, and ξj are slack variables that account for the soft-margin misclassification of observations. There were different kernel functions evaluated in this study including (1) the linear kernel (K(x, x′) = x·x′), (2) the polynomial kernel (K(x, x′) = (*γ*x·x′ + r)d), (3) the radial basis function (RBF) Gaussian kernel (K(x, x′) = exp.(−γ||x − x′||^2^)), and (4) the cubic kernel which is a polynomial of degree d = 3. To find the best-performing model configuration, systematic grid search techniques were used to improve the box constraint parameter C (where C∈{0.1,1,10}) and kernel scaling parameters inside the cross-validation paradigm.

#### 3.5.2 K-Nearest Neighbors (KNN)

The k-Nearest Neighbors (KNN) classifier assigns a label to a query sample using majority vote from the k nearest neighboring samples. The KNN classifier is defined as: ŷ = argmax_c ∑_(x_i ∈ N_k(x)) I(y_i = c) where N_k(x) is the set of k nearest neighboring samples (x) according to a metric d(·, ·) and I(·) = an indicator function which equals 1 if x belongs to class c, equals zero otherwise. This study evaluated several distance metrics including: Euclidean distance defined as d(x, x’) = ||x - x’||₂; cosine distance defined as d(x, x’) = 1 - (x · x’)/(||x|| ||x’||); and Minkowski distance with *p* = 3 (cubic distance). The sizes of neighborhoods that were evaluated for their influence on classification performance were k ∈{1, 10, 100}. Variations on this “weighted” version of KNN were also implemented, where a neighbor was weighted inversely proportional to its distance. Thus, in the construction of the final classification decision, samples closer to the query sample exerted greater influence than those farther away.

#### 3.5.3 Subspace KNN (an ensemble method)

The subspaces method is an ensemble approach, which consists of training multiple base learners, in our case the KNN classifiers, using random selections of features. In our experiment, we used the Subspaces KNN algorithm, where 30 KNN classifiers were trained using 17 randomly selected features. Using Subspace KNN learning methods may yield more accurate predictions or classifications than using a single learner, as they combine predictions or classifications from multiple learners to produce a single prediction or classification. In bagging (bootstrap aggregation), M base learners will model the dataset with M separate bootstrap samples of the data points. The output from these M learners can then be pooled using majority voting to get a single output prediction or classification for each input sample:

$$\hat{y}=\text{mode}\{h₁(x),h₂(x),\ldots ,h\_M(x)\}.$$

where h_m(x) is the prediction made by the mth base learner on the input sample x.

On the other hand, boosting is an incremental algorithm that trains a number of weak classifiers, where the last-trained weak classifier gives more importance to instances that were wrongly classified by earlier weak classifiers. The final classifier is thus defined as:

H(x)=sign(∑_(m=1)∧Mα_mh_m(x)).

where the weight of the mth weak learner is defined as:

α_m=½ln((1−ε_m)/ε_m).

and where ε_m is the weighted error of the mth weak learner. Subspace KNN were also examined using models trained on randomly selected feature subsets to increase the diversity of the base learners, thereby decreasing correlation and reducing the tendency for each base learner to overfit its respective bootstrap sample of the training data. Although the RUSBoost algorithm allows random undersampling of the majority class at each subsequent boosting iteration to correct for class imbalance, it does not apply to this study because the dataset is balanced. Using decision trees as base learners with maximum splits of 4, 20, or 100, three ensemble sizes (M ∈ {30, 50, 100}) are evaluated to determine which configuration yields the best results.

#### 3.5.4 Neural networks

The Adam optimizer was used to train this model with a learning rate of 0.001 and categorical cross-entropy as the loss function. Training was completed without any early stopping, dropout, or regularization (*λ* = 0) and used a maximum of 1,000 epochs so that the model could be trained without limitations. To promote stable and efficient convergence during training, the weights were initialized using Xavier initialization, and the batch size was equal to the entire dataset to provide consistent gradients with each iteration during training. Fully connected feed-forward neural networks with ReLU activation functions were used for multi-class classification. For each feed-forward network, defined as a neural network with L layers, the forward propagation of data through the network is defined as follows:

a^(l)=ReLU(W^(l)a^(l−1)+b^(l)),wherelranges from1throughL−1.

y^=softmax(W^(L)a^(L−1)+b^(L)).

where ReLU(z) = max(0,z). W^(l) and b^(l) will represent the weight matrix and bias vector for layer l, respectively. The output layer will use the softmax function to produce class probabilities for all possible classes. Five different architectures were tested including (1) Narrow (1 hidden layer of 10 neurons), (2) Medium (1 hidden layer of 25 neurons), (3) Wide (1 hidden layer of 100 neurons), (4) Bi-layered (2 hidden layers of 10 neurons), and (5) Tri-layered (3 hidden layers of 10 neurons). The models were trained without regularization (*λ* = 0) using the Adam optimizer, categorical cross-entropy loss, and a learning rate of 0.001 for a maximum of 1,000 iterations. The complete model training and evaluation procedure is summarized in Algorithm 1.

#### 3.5.5 Additional algorithms

The models developed to assess this classification included additional algorithms to provide a comprehensive understanding of the comparison. For the Decision Trees, splittings at the maximum values of 4, 20, and 100 were used to create partitions in the nodes with the Gini Impurity measure. Discriminant Analysis was performed using Linear (LDA) and Quadratic (QDA) in accordance with the assumption of Multivariate Normal Class Conditional Distributions. Naive Bayes was performed using the Gaussian and Kernel Density Estimation methods. Kernel-based techniques, including Kernel SVM and Kernel Logistic Regression, were implemented using Random Fourier Feature Approximations (also known as Density Estimates) for increased computational efficiency. Efficient Logistic Regression and Efficient Linear SVM are two additional efficient model types that offer large-scale, efficient solutions by reducing the computational complexity and having competitive prediction capabilities.

### 3.6 Model training and hyperparameter optimization

All models were trained and assessed using 10-fold cross-validation on the training set, yielding 33 models. The dataset was split into 10 separate folds (F₁, F₂, …, F₁₀) of roughly equivalent size. For each fold (j), the model would be trained on the other 9 folds and validated on fold (j), with the accuracy of the validation being expressed mathematically as follows:

Acc_CV=[1/10Σ(j=1to10)Accuracy(M,F_j)].

Where M is the model that was created using the other 9 folds excluding fold (j), this process was performed multiple times for each hyperparameter configuration in the scope of a grid search, and the configuration with the highest cross-validation accuracy was chosen. The final optimal hyperparameter set was then used to train the final model on a full dataset consisting of n = 1,301, and the holdout set was used only for final evaluation to ensure that the performance estimates were independent of model selection and hyperparameter tuning.

### 3.7 Performance evaluation metrics

Classification accuracy is the primary performance criterion and is calculated as follows:

Accuracy=(Correct Predictions)/(Total Predictions).

In the multi-class situation, Accuracy indicates what proportion of its test samples have been correctly predicted by the model. Along with calculating total accuracy we also calculated metrics that provided performance information on a per-class basis:

Precision_c=TP_c/(TP_c+FP_c).

Recall_c=TP_c/(TP_c+FN_c).

$$\begin{array}{l} F1-\text{score}\_c=(2\times \text{Precision}\_c\times \text{Recall}\_c)\\ /(\text{Precision}\_c+\text{Recall}\_c). \end{array}$$

Where c is for classes {−1, 0, +1}; TP’s are true positives, FP’s are false positives and FN’s are false negatives. We calculated macro-averaged metrics (unweighted mean across all classes) and weighted averages (taking into consideration the class imbalance). The level of agreement beyond chance was evaluated using the Cohen’s Kappa statistic. In order to quantify uncertainty associated with these measures, we calculated 95% confidence intervals via stratified bootstrap resampling (1,000 iterations) of class proportions in each bootstrap sample.

### 3.8 Statistical analysis and model comparison

Model generalization was evaluated by comparing the test and validation accuracies of models produced using K-fold cross-validation. Absolute differences between the two measures (|Acc_validation − Acc_test|) of less than 2% were rated as excellent, between 2 and 5% as good, and any larger indicated possible overfitting/shifted dataset, or generalization failure. The Pearson correlation coefficient was used to evaluate the relationship between validation and test performance across all 33 models, and individual model families (SVM, KNN, etc.) were summarized descriptively by mean, standard deviation, min/max, and accuracy range. Accuracy in the final model(s) selected based on test set accuracy, and, in addition to that, at times, certain computational efficiencies were considered before determining whether they would be able to deploy in practice successfully.

Algorithm 1 Complete Training and Evaluation Pipeline.**Input**
: Dataset D with N=1626 samples, p=34 features, and target y ∈ {-1,0,1}
**Output**: Best model M* with performance metrics
1:  **# Data Preprocessing**
2:  Standardize continuous features: z_i ← (x_i - μ) / σ
3:  One-hot encoding for categorical features
4:  Check data completeness (no missing values)
5:  **# Partitioning datasets**
6:  Split data into two sets using Stratified Split to create D_train (n=1301, which is 80%) and D_test (n=325,
     which is 20%)
7:  From D_train create 10 Stratified Folds (i.e. F1, F2, …, F10)
8: **# Training of Model**
9: Create List of Models to be Trained = [Trees, SVM, KNN, Subspace KNN, NN] (total of 33 models)
10: Create Empty List of Results for All Models
11: **For** Every Model Family M in the List of Models
12: **For** each model configuration θ:
13:  Initialize cumulative cross-validation accuracy acc_cv = 0
14:  **# Perform 10-fold cross-validation**
15:  **For** fold j = 1 to 10:
16:   Define training subset as D_train, excluding Fj
17:   Define validation subset as Fj
18:   Train model Mjusing (M, θ) on the training subset
19:   Add validation accuracy to acc_cv
20:  **End For**
21: Calculate mean cross-validation accuracy: acc_cv = acc_cv / 10
22: **End For**
23: Determine the best hyperparameter set θ* based on maximum mean cross-validation accuracy
24: Train the final model M_final with θ* on the entire D_train data
25: Test M_final on D_test to get test accuracy acc_test
26: Calculate other performance measures such as computational cost, training time, and prediction speed
27: Append (M, θ*, acc_cv, acc_test, cost, time, speed) to the list of results
28: **End For**
29: **# Model Selection**
30: Find M* as the model with the highest test accuracy among all models.
31: Output M* and the entire set of results

**Figure 1:**
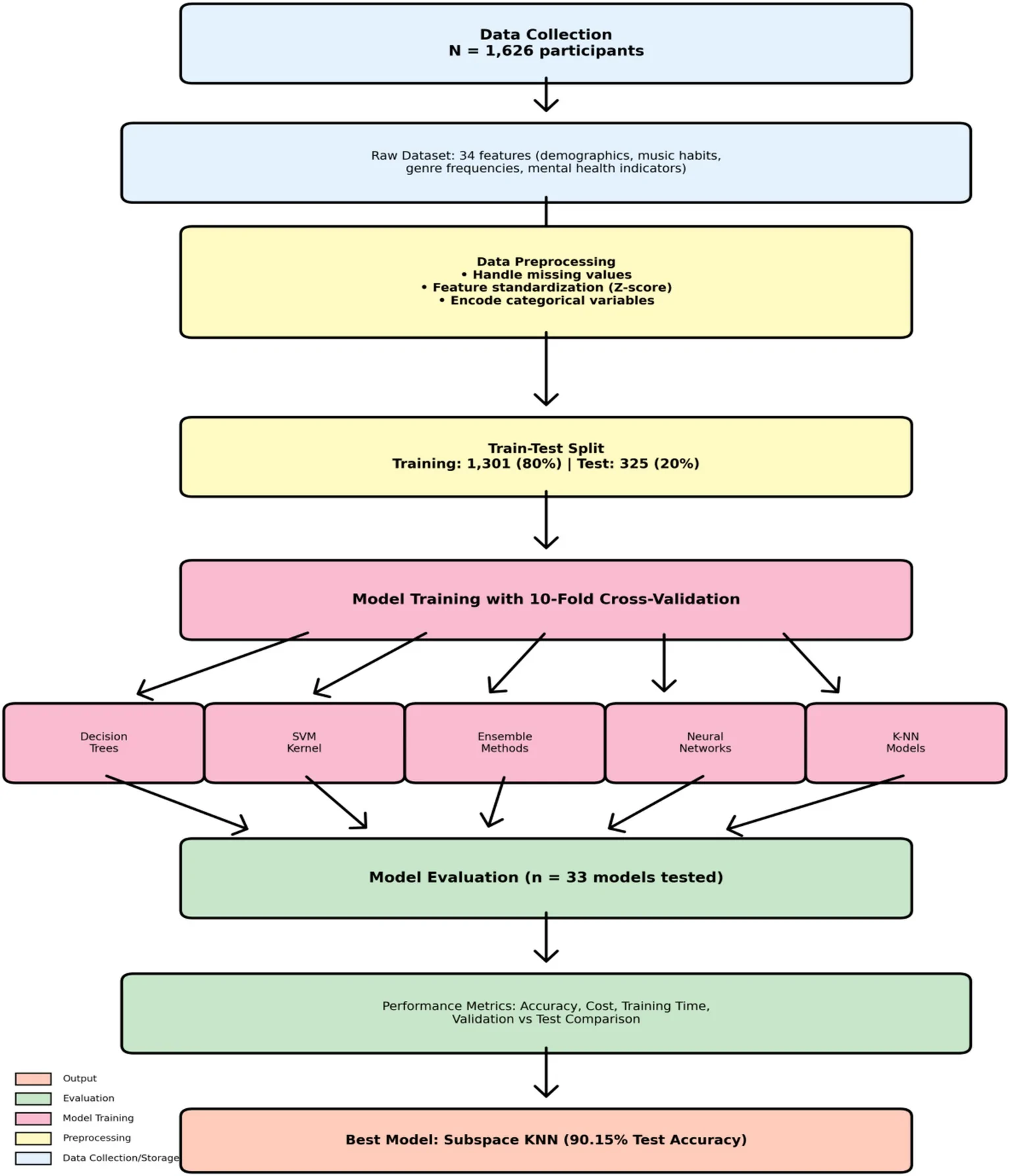
Methodology flowchart.

Figure 1 illustrates the methodological process undertaken in the study, from data collection with *N* = 1,626 participants to preprocessing and feature standardization. The data is split into 80/20 stratified train-test sets, and further validated using 10-fold cross-validation on the training data. A total of 33 models from five algorithm families—Decision Trees, SVM, KNN, Subspace KNN Methods, and Neural Networks—are trained and validated, and the final model is selected based on test accuracy. The color coding in Figure 1 represents blue (data collection/storage), yellow (preprocessing), pink (training), green (evaluation), and orange (output).

### 3.9 Software and computational environment

All analyses were performed in MATLAB R2023a (The MathWorks, Inc.) on Windows 11 Pro (Intel Core i7-12700K, 32GB RAM). A constant random seed of 42 was set in all the stochastic operations (rng(42, ‘twister’)). For the best-performing Subspace KNN, the final hyperparameter values were: number of learners = 30, number of predictors per learner = 17, K neighbours = 10, Euclidean metric, and KNN base learner. Stratified 10-fold cross-validation was programmed in the MATLAB environment using the cvpartition function. Data preprocessing steps included: (1) verification of the presence of missing values (not present), (2) one-hot encoding of the categorical features (streaming service and favorite genre), and (3) Z-score standardization (*μ* = 0, *σ* = 1) of the continuous features using the training set statistics and then the test set. The Kaggle dataset used in this study is accessible to the public at https://www.kaggle.com/datasets/catherinerasgaitis/mxmh-survey-results.

## 4 Experimental results

This study is not the first to show that there is no one best machine learning algorithm, but it is the methodological lesson learned from sensitivity analysis that is the principle scientific contribution. Subspace KNN performed best in terms of classification accuracy; removing baseline mental health variables led to a significant drop in the accuracy of the model, showing that the composition of predictors is much more important than the algorithm for the accuracy of the model. This is consistent with a new paradigm for data-driven AI, one in which simply improving the quality of the data and engineering features and measurements can have a more significant impact than fine-tuning learning algorithms. Therefore, the results of the present study are best viewed as evidence of the importance of careful study design and predictor selection in the prediction of behavioral health outcomes rather than as proof of the superiority of any specific classifier. While 33 machine learning algorithms were tested in this research, the main scientific value of the research is not the selection of the single best performing algorithm. Instead, the study illustrates that the choice of predictors, the composition of features and the experimental design are more influential on predictive performance than the choice of algorithm. This is an observation that reflects the current trend toward data-centric Artificial Intelligence, where incremental improvements in the quality of data, feature engineering and measurement protocols can result in a more significant improvement than incremental improvements in learning algorithms. Thus, the present work offers methodological direction for future behavioral health predictive studies not covered by the context under consideration.

While it is well known that music can have a positive effect on mental wellbeing, around a third of the participants in the current study said music had a negative effect on their mental wellbeing. The findings of this study should be treated with caution because of its study design, which does not allow for causal inference. However, there were a number of potential explanations in previous studies. For people with anxiety or depression, music that is congruent with their mood may be particularly appealing, drawing them to the melancholy and negative emotions associated with it and encouraging negative mood rumination. Additionally, music can trigger negative emotional reactions, even in the absence of psychological relief, if music is used to evoke distressing personal memories, or if the music is evoked by adverse life events. It is also possible that emotional reactions, temperaments, cultural backgrounds, and psychological susceptibility can affect the perception of the music’s positive or negative impact. Thus, the reactions that were observed may be viewed as evidence of a multifactorial effect of music and listener, and not necessarily as an overall negative reaction to music.

The results support the trend toward data-based approaches to machine learning, showing that progress in terms of predictors and design of experiments often yields more than advances in classification algorithms.

### 4.1 Participant characteristics and mental health prevalence

**Figure 2:**
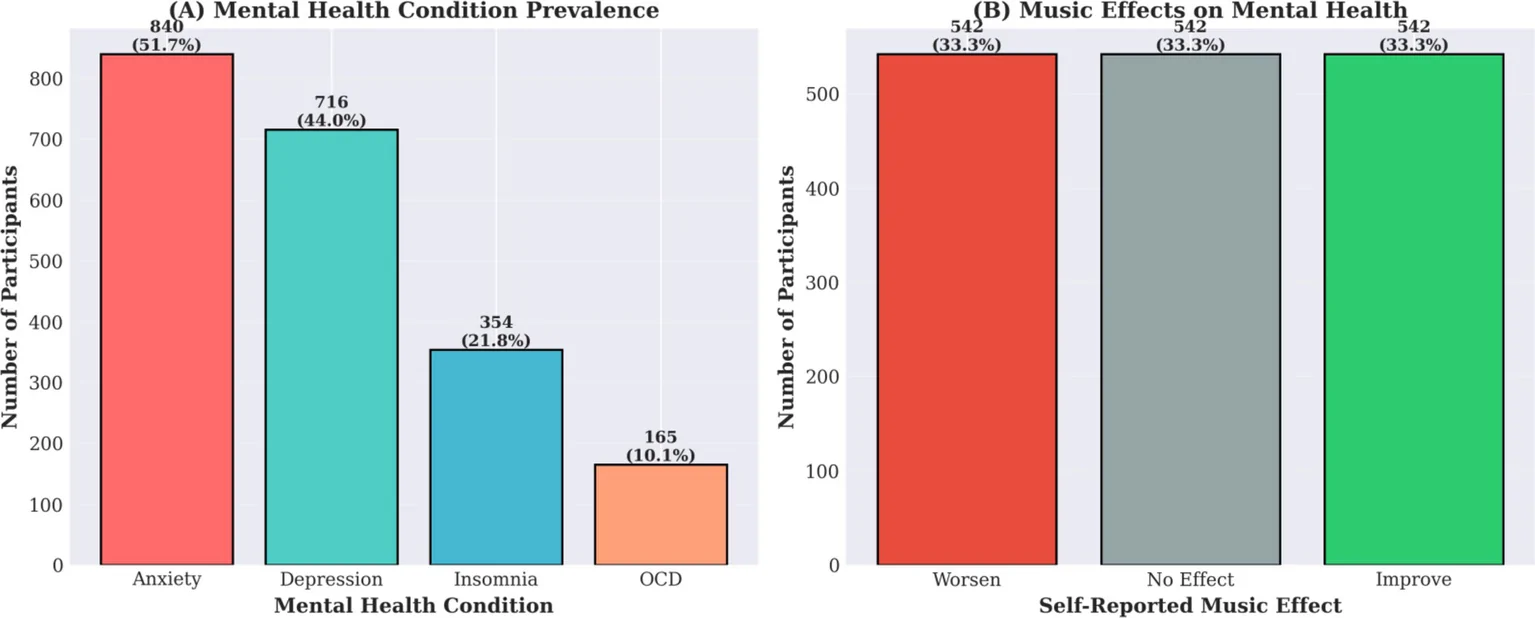
Distribution of **(A)** mental health conditions and **(B)** perceived effects of music on mental health.

**Figure 3:**
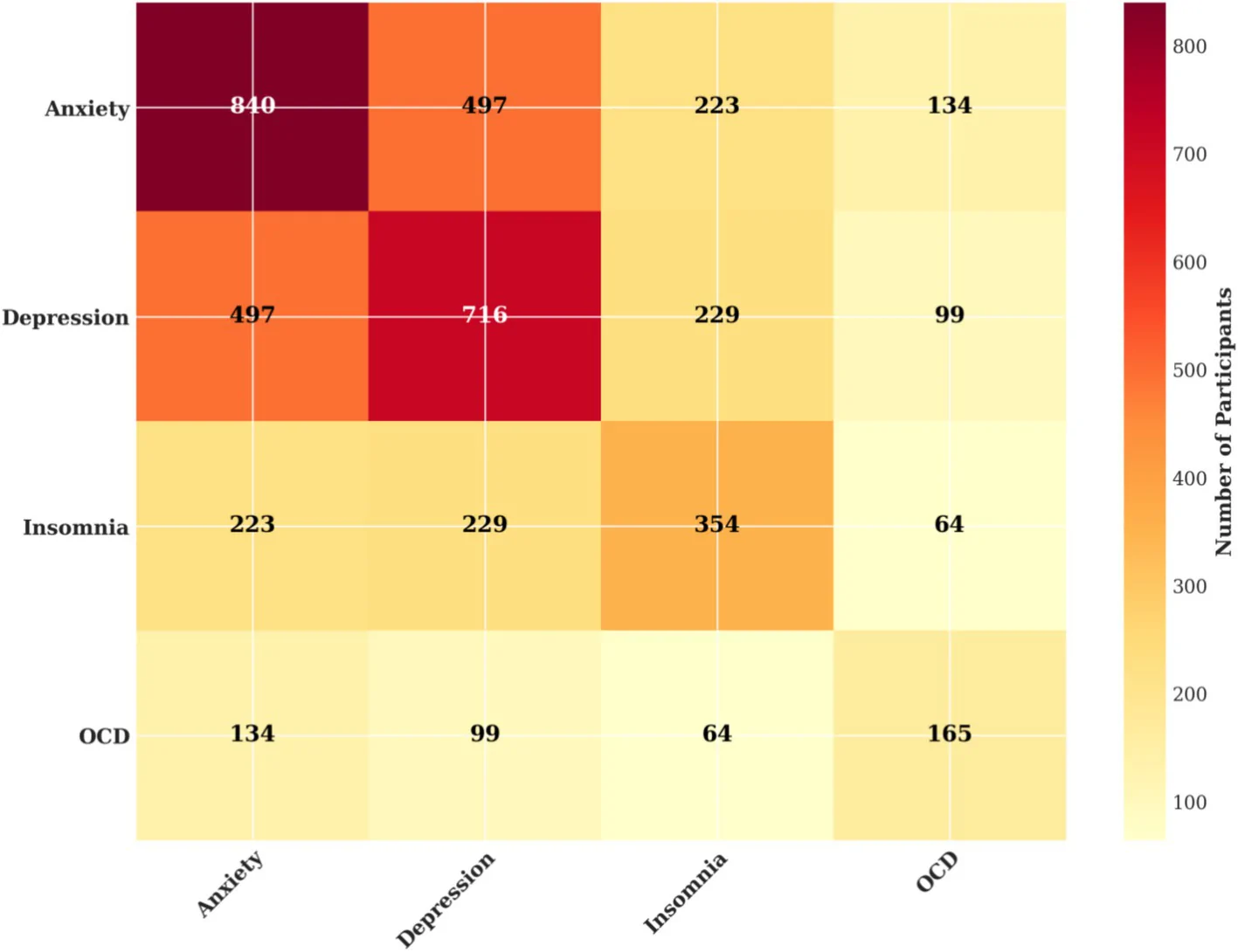
Mental health condition comorbidity matrix.

The study included 1,626 participants, as depicted in Figure 2A. Participants in this sample had a high prevalence of mental health disorders; thus, it is clinically relevant to examine the possible role of music as an intervention for people in psychological distress. Specifically, there were 840(51.7%) participants who reported an experience with anxiety; 716 participants (44.0%) reported an experience with depression; 354 (21.8%) reported an experience with insomnia; and 165 (10.1%) reported an experience with obsessive-compulsive disorder. These results are summarized in Table 4. There were also significant rates of comorbidity, providing further evidence of how mental health disorders overlap within the sample population, as shown in Figure 3. Of the participants who reported anxiety, 497 (59.2%) also reported an experience with depression, illustrating an association between the two disorders; 223 (26.5%) of the participants with anxiety also reported insomnia, which is consistent with research demonstrating the connection between anxiety and sleep difficulties. Lastly, the present findings continue to demonstrate the interrelated and complex nature of the mental health profiles among the sample participants and provide an opportunity to implement music-based interventions.

**Table 4:** Mental health condition prevalence and comorbidity patterns (*N* = 1,626).

Characteristic	N	Percentage (%)	Comorbidity with anxiety (%)
Anxiety	840	51.7	100.0
Depression	716	44.0	59.2
Insomnia	354	21.8	26.5
OCD	165	10.1	16.0

### 4.2 Music listening behaviors and engagement patterns

According to music engagement behaviors, most participants in the sample reported frequent, diverse interactions with music in their everyday activities, as shown in Table 5. For instance, 969 individuals (59.6%) reported listening to music while performing work duties. This suggests that music plays a role, at least to some degree, as a secondary task when performing regularly scheduled tasks (or “everyday activities”). A large number of participants were also actively creating music; 273 participants (16.8%) identified themselves as instrumentalists, while 135 (8.3%) identified as composers, suggesting they were deeply involved in the creative process of making music. Additionally, 865 participants (53.2%) had actively searched for new musicians or music genres, indicating that participants’ music consumption was diverse and exploratory. The participants exhibited a wide range of music engagement behaviors, from listening to everyday music to creating a piece of music and seeking out or discovering new musicians and genres.

**Table 5:** Music listening behaviors and engagement patterns among participants (*N* = 1,626).

Music behavior	N	Percentage (%)
Listen while working	969	59.6
Instrumentalist	273	16.8
Composer	135	8.3
Explore new music	865	53.2
Listen to foreign languages	731	45.0

### 4.3 Self-reported music effects on mental health

Three groups of subjects were created based on self-reported data regarding the impact of music on their mental well-being, as indicated in Figure 2B. The distribution of the cohort was exactly symmetrical across all three outcomes (i.e., “Music has improved my mental well-being,” “Has had no effect on my mental well-being” and “Music has made my mental well-being worse”), with a total of 1,626 subjects represented (0.3333 or 33.33% reported each outcome) as shown in Table 6. Equal proportions of the three outcome classifications provided the researchers with a perfect dataset for machine learning classifier creation, since it provided sufficient samples for each classification and eliminated class imbalance problems that negatively impact models during model development, model evaluation, and generalizability across populations.

**Table 6:** Distribution of self-reported music effects on mental health (*N* = 1,626).

Music effect category	N	Percentage (%)
Worsen (−1)	542	33.3
No Effect (0)	542	33.3
Improve (+1)	542	33.3

### 4.4 Genre preferences and listening frequency patterns

**Figure 4:**
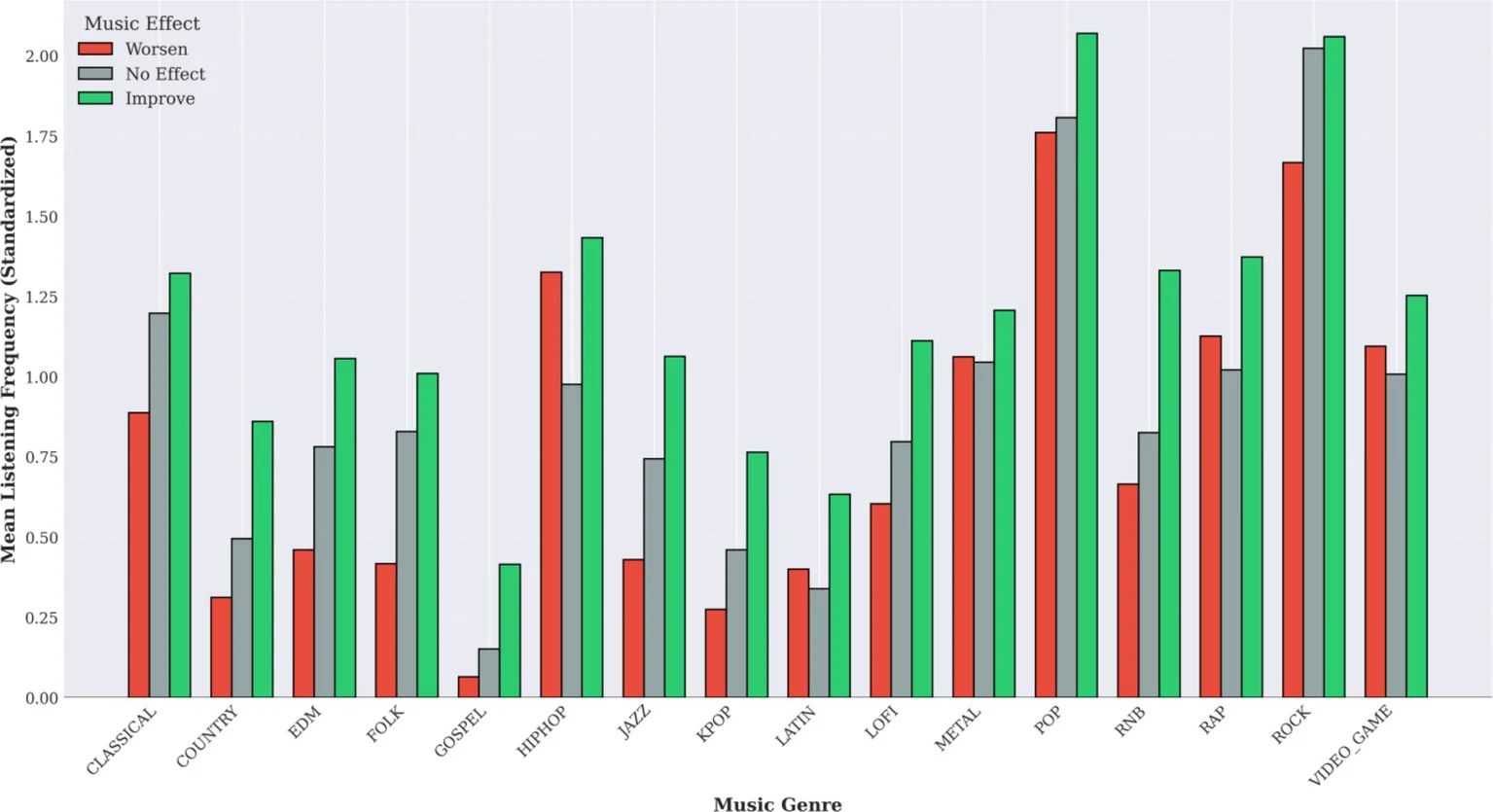
Genre listening frequency patterns by perceived music effect on mental health.

Distinct musical preferences were identified within groups of participants who reported different effects of music on their mental health, as shown in Figure 4. Mean listening frequencies for specific genres of music perceived to improve mental health (i.e., classical, jazz, and lofi) tended to be higher than mean listening frequencies for music genres perceived to worsen mental health (i.e., metal and rap). Given the apparent connection between musical genre and emotional and cognitive responses to music, genre selection may mediate the effects of music on mental health. However, because the dataset is cross-sectional, it is not possible to determine the exact direction of causality of the observed patterns. It is unclear whether listening to a specific genre improves or worsens mental health, or whether a person’s emotional state at the time of listening dictates their musical genre preferences. In summary, individual differences in musical preferences can provide insight into the roles of personality characteristics, emotional coping styles, and other contextual/situational factors in shaping how a person consumes music and their mental health.

### 4.5 Machine learning model performance and comparison

**Figure 5:**
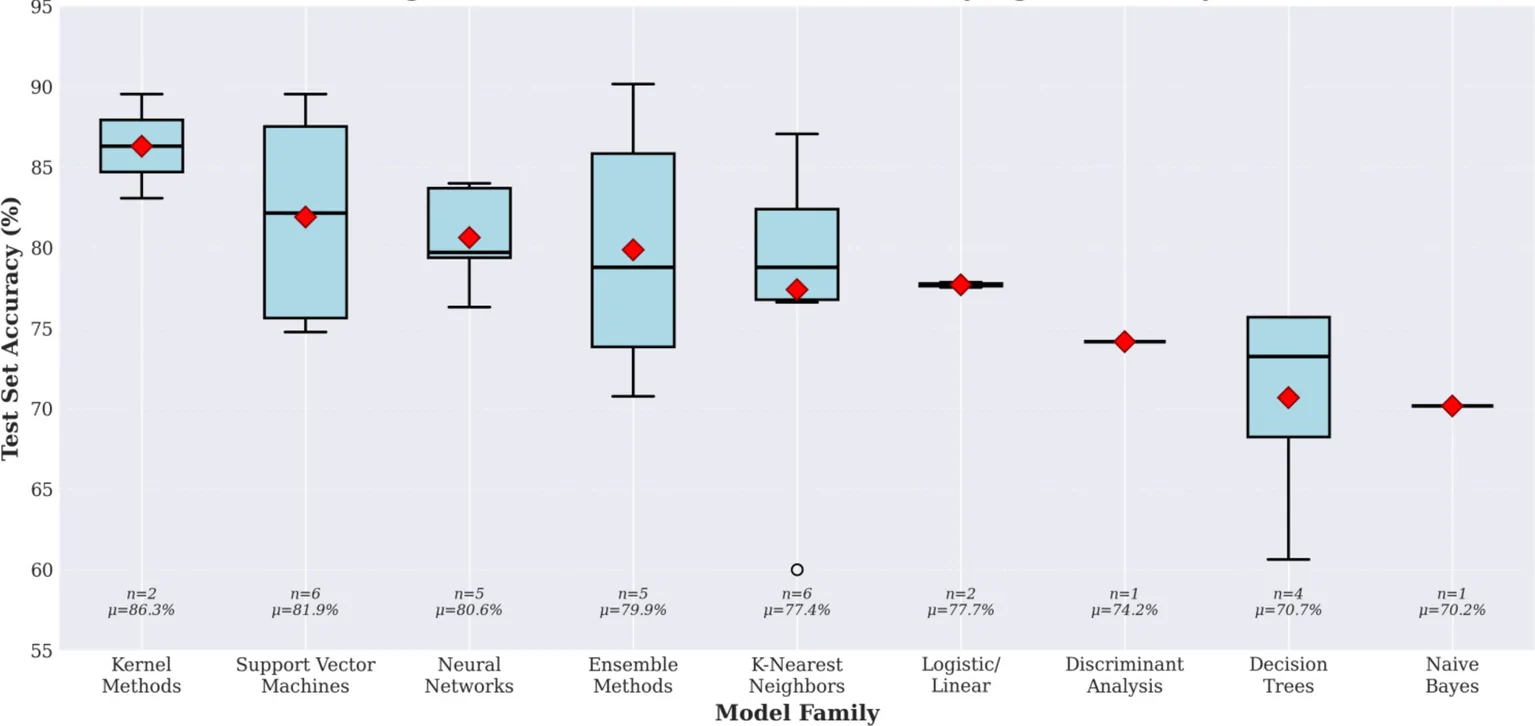
Model performance distribution by algorithm family.

Of the 33 models trained, 32 ran successfully. One model (Tri-layered Neural Network) did not converge and was not considered for further analysis. An extensive assessment of 32 machine learning methods was carried out using an exhaustive 10-fold cross-validation scheme applied to a total of 1,301 training instances, with final model performance evaluated against an Independent held-out test set of 325 cases. The set of assessed machine learning algorithms included a variety of algorithmic families (e.g., Decision Trees, Support Vector Machines, K-Nearest Neighbors, Subspace KNN methods, Neural Network models, Discriminant Analysis models, and Kernel-based methods), as shown in Figure 5. Each of the algorithms was trained using a shared and complete feature set, which comprised thirty-four independent variables that were collected from each participant to characterize important areas of participant demographics, music listening behaviors, frequency of listening to specific music genres, and tempo preferences in beats per minute; as well as indicators of self-reported mental health, all of which were provided in Table 3. As a result of this comprehensive modelling approach, each algorithm could be effectively compared across a diverse set of learning models, while ensuring access to the same information for Multidimensional Predictive Learning.

**Figure 6:**
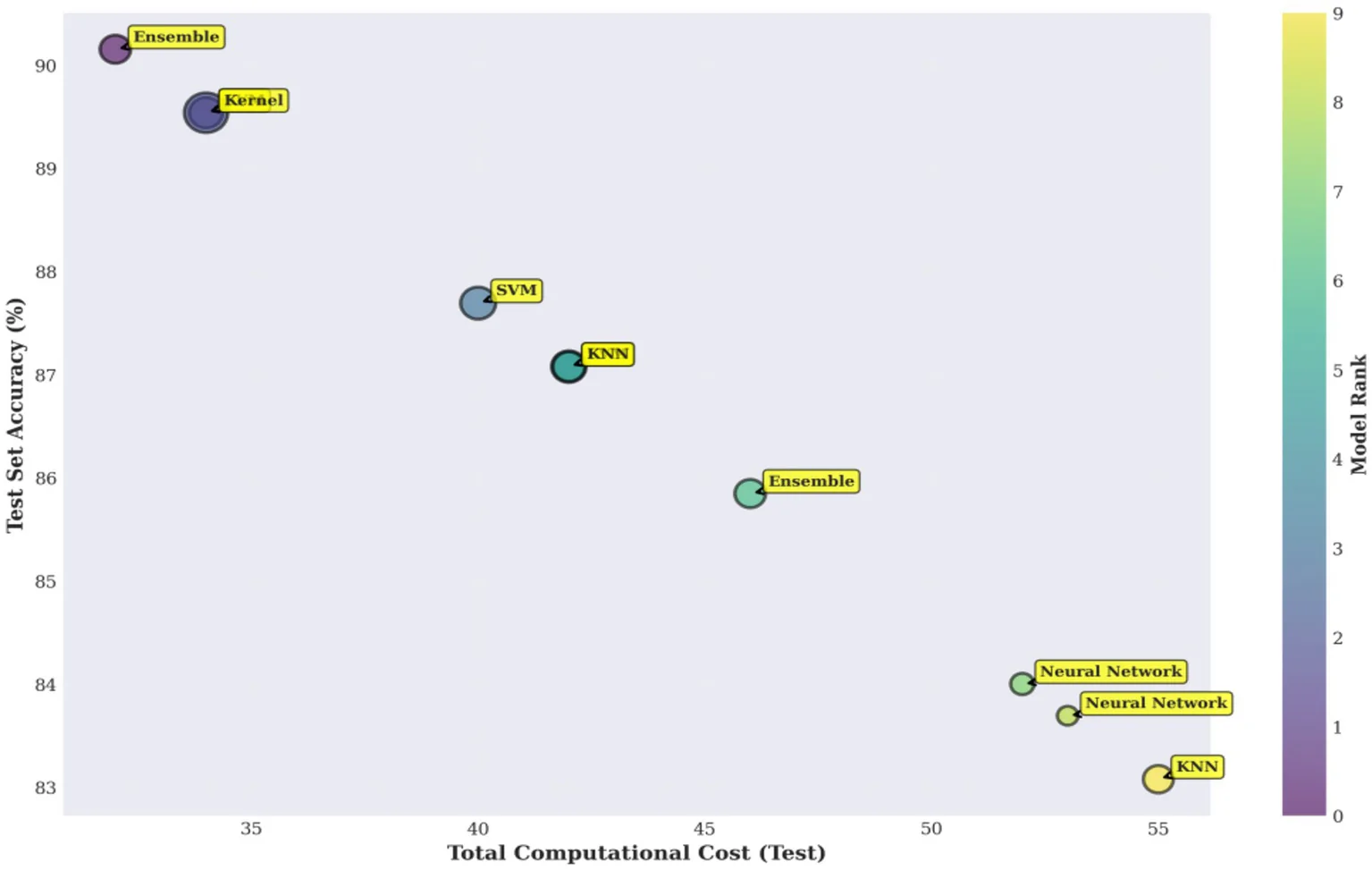
Top 10 models- accuracy vs. computational cost.

The best-performing model of the different models used was the Subspace KNN model. The results show that the performance of the Subspace KNN model (Model 2.25) was 90.15% (test set) and validation of 88.39%. The results indicate strong generalization and little evidence of overfitting, as shown in Figures 5, 6. The 1.76% difference between validation and test was a strong indication of stable, robust model performance and a good predictor of new or previously unseen data. The model results remained the highest-performing among all other models, as shown in Figure 6. It achieved the highest test accuracy of 90.15% and a validation accuracy of 88.39%. The next model, Bagged Trees, achieved an test accuracy of 85.85% and a validation accuracy of 85.09%. The third-highest-performing model, Boosted Trees, achieved test accuracy of 78.77% and validation accuracy of 75.86%. Subspace KNN methods may achieve high predictive performance by combining multiple weak learners using techniques such as bagging, boosting, and subspace sampling. Together, these methods can reduce variability, help prevent overfitting, and enable modeling of complex nonlinear relationships between music listening behavior and mental health outcomes. In particular, one reason the Subspace KNN method is successful is that it trains multiple KNN classifiers on randomly selected subsets of the feature data. This method leveraged the many predictor variables that constitute the large, high-dimensional predictor space while helping overcome the curse of dimensionality, as demonstrated in Table 7.

**Table 7:** Top 15 performing machine learning models ranked by test set accuracy.

Rank	Model type	Model family	Val Acc (%)	Test Acc (%)	Cost	Time (s)
1	Subspace KNN	Subspace KNN Methods	88.39	90.15	32	10.05
2	SVM	Support Vector Machines	86.70	89.54	34	11.94
3	Kernel	Kernel Methods	86.86	89.54	34	19.94
4	SVM	Support Vector Machines	84.55	87.69	40	13.04
5	SVM	Support Vector Machines	86.78	87.08	42	12.63
6	KNN	K-Nearest Neighbors	85.17	87.08	42	10.92
7	Subspace KNN	Subspace KNN Methods	85.09	85.85	46	10.08
8	Neural Network	Neural Networks	83.55	84.00	52	5.79
9	Neural Network	Neural Networks	81.71	83.69	53	4.54
10	KNN	K-Nearest Neighbors	81.17	83.08	55	9.56
11	Kernel	Kernel Methods	82.78	83.08	55	13.08
12	KNN	K-Nearest Neighbors	78.02	80.31	64	8.51
13	Neural Network	Neural Networks	79.63	79.69	66	11.52
14	Neural Network	Neural Networks	77.56	79.38	67	14.42
15	Subspace KNN	Subspace KNN Methods	75.86	78.77	69	20.38

Val Acc, Validation accuracy (10-fold CV); Test Acc, Test set accuracy; Cost, Total computational cost; Time, Training time in seconds.

The performance metrics for the Subspace KNN model showed an average classification accuracy of 90.15% on the test data set. The model showed good inter-class consistency with Cohen’s Kappa statistic (K = 0.852) and Matthews Correlation Coefficient (MCC = 0.853). Precision, recall, and F1-scores for individual classes showed values above 0.89 for all three classes (Worsen, No Effect, Improve). The results in Table 8 showed stable model performance. The confidence intervals for the performance metrics were obtained using stratified bootstrap resampling (1,000 iterations). Macro and weighted averages for the performance metrics were the same at 0.902.

**Table 8:** Detailed classification metrics for subspace KNN.

Metric.	Worsen (−1)	No effect (0)	Improve (+1)	Macro average	Weighted average
Precision	0.896	0.907	0.903	0.902	0.902
Recall	0.899	0.898	0.909	0.902	0.902
F1-Score	0.897	0.902	0.906	0.902	0.902
Support	69	108	148	325	325

**Figure 7:**
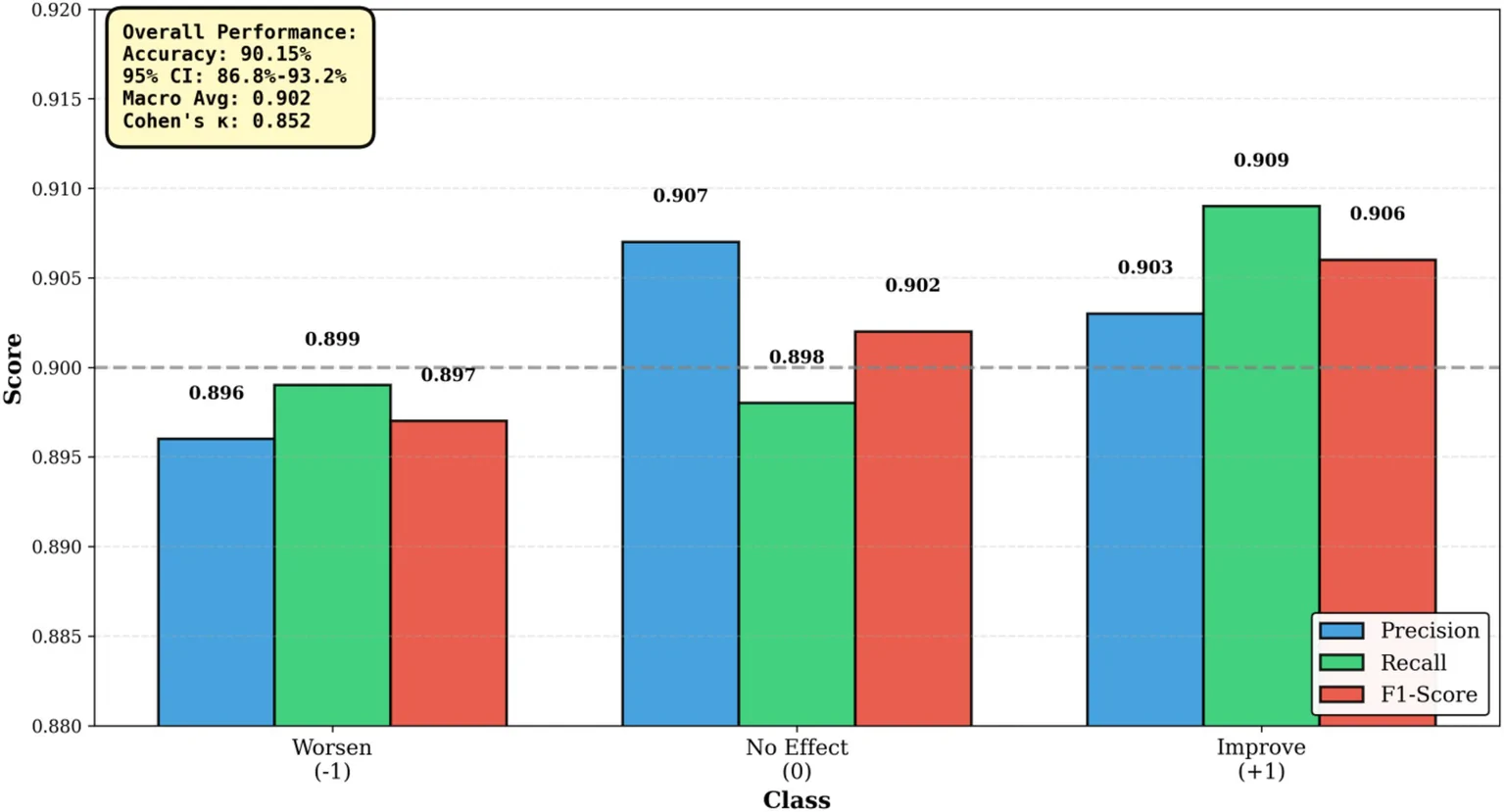
Performance metrics comparison across classes (Subspace KNN model).

The Figure 7 below depicts a comparative analysis of Precision, Recall, and F1-score for three different classes: Worsen (−1), No Effect (0), and Improve (+1), with all scores ranging from 0.896 to 0.909. In addition, the Improve (+1) class obtained the highest recall and F1-score of 0.909 and 0.906, respectively, while the No Effect class obtained the highest precision of 0.907. Moreover, the Worsen class obtained balanced scores ranging from 0.897 to 0.899. An accuracy of 90.15% was obtained, with a 95% CI of 86.8 to 93.2%, a macro average of 0.902, and a Cohen’s κ of 0.852.

**Figure 8:**
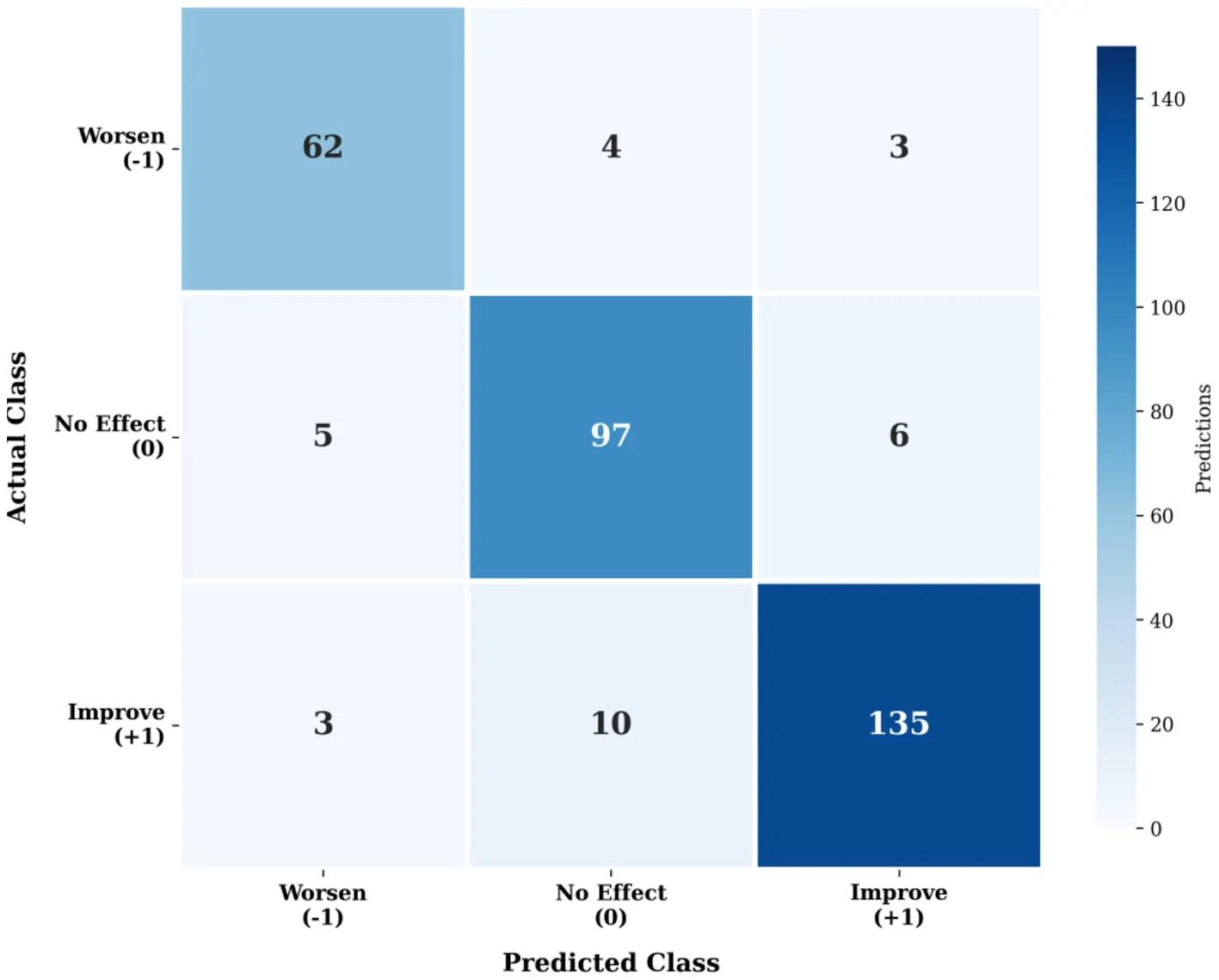
Confusion matrix [Subspace KNN model, test set (*N* = 325)].

The Figure 8 shows the confusion matrix of three classes: Worsen (−1), No Effect (0), and Improve (+1), where there is strong classification accuracy with most of the predictions concentrated on the diagonal line. The model’s strong predictions include 62 for Worsen, 97 for No Effect, and 135 for Improve, indicating strong performance in the Improve class. The misclassified predictions have low occurrences: 4 and 3 from the Worsen class, 5 and 6 from the No Effect class, and 3 and 10 from the Improve class.

#### 4.5.1 Comparison with baseline models

For the comparative assessment of the effectiveness of the proposed algorithm Subspace KNN (accuracy = 90.15%), we considered the following baseline approaches: (1) a chance level baseline of 33.3%, when all class probabilities were equal; (2) a majority class baseline of 33.3%, which, for a perfectly balanced three-class problem, was also equal to random chance; (3) a Logistic Regression model that provided an accuracy of 76.3%; and (4) results obtained by previous studies. Prior works using musical preferences as predictors of mental state showed the accuracy rates in the range between 32 and 76% (32, 33). For instance, study (32) used the gradient boosting approach for predicting the anxiety state using genre preferences in music, obtaining an accuracy of 32%. Another study (33) proposed a random forest classification algorithm to predict depression, anxiety, and insomnia based on music listening, reaching an accuracy rate of 76.35%. The suggested model demonstrates a better result than the highest accuracy rate reported earlier, which is 13.80 points higher (76.35%). In addition, the model is far superior to random and majority baselines by 56.85 points. As mentioned above, for a balanced dataset, the majority and random baselines are equal.

### 4.6 Model family performance comparison

Based on our aggregate analysis across the various model families, we found considerable variance in predictive capabilities between the families, as illustrated in Table 9. Kernel Methods had the highest mean test accuracy (86.31%), followed by Support Vector Machines (81.90%) and Neural Networks (80.62%), with kernel-based models and support vector machines the next highest. The hierarchical performance of each model family highlights some of the fundamental differences between the ability of different algorithmic paradigms to model the true underlying structure of the data. More specifically, ensembles leverage a collection of individual learners to semi-accurately model complex, nonlinear relationships, thereby decreasing the potential for variance and overfitting. Similarly, kernel-based and support vector machines achieve high performance because they can model nonlinear decision boundaries in high-dimensional feature spaces. These results indicate that choosing model families that are representative of the complexity and heterogeneity that exists in behavioral and mental health datasets is critical.

Using a variety of kernel functions within support vector machine (SVM) models consistently yielded strong, competitive results, as shown in Figure 6. Among the SVM kernel models used, both the medium Gaussian SVM and the SVM kernel model achieved the highest test accuracy of 89.54%. In comparison, the quadratic and cubic SVMs had test accuracies of 87.69 and 87.08%, respectively, as shown in Table 9. All kernel-based techniques demonstrated strong effectiveness in capturing the highly nonlinear and complex relationships between music-related features and their associated mental health outcomes. Among the kernels used, the Gaussian radial basis function kernel is particularly effective at creating a linear decision boundary in an infinite-dimensional, feature-based metric space from the original metric input data, thereby allowing highly nonlinear associations between the predictors and outcomes.

**Table 9:** Summary statistics of test set accuracy by model family.

Model family	N	Mean Acc (%)	SD	Min (%)	Max (%)
Kernel Methods	2	86.31	4.57	83.08	89.54
Support Vector Machines	6	81.90	6.90	74.77	89.54
Neural Networks	5	80.62	3.23	76.31	84.00
Subspace KNN Methods	5	79.88	8.09	70.77	90.15
Logistic/Linear Models	2	77.69	0.22	77.54	77.85
K-Nearest Neighbors	6	77.38	9.36	60.00	87.08
Discriminant Analysis	1	74.15	nan	74.15	74.15
Decision Trees	4	70.69	7.11	60.62	75.69
Naive Bayes	1	70.15	nan	70.15	70.15

N, number of models; Mean Acc, mean test accuracy; SD, standard deviation; Min/Max, minimum and maximum test accuracy within family.

**Figure 9:**
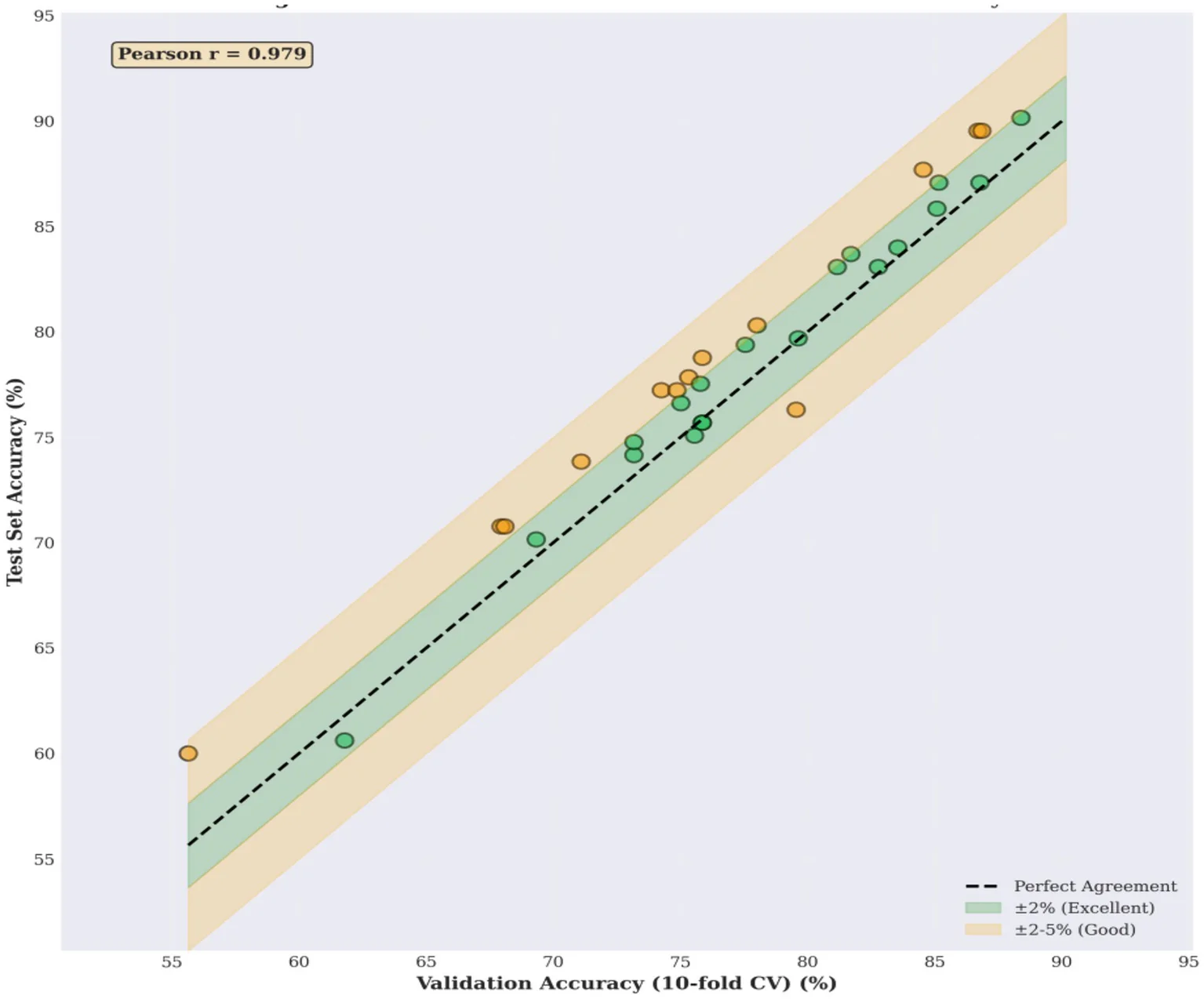
Model generalization- validation vs. test accuracy.

Results from neural network architectures show that their architectures exhibit wide performance variability. The Wide Neural Network (100 hidden units) achieved the highest test accuracy of 84 percent. It was closely followed by the Medium Neural Network (25 hidden units) at 83.690 percent and the Narrow Neural Network (10 hidden units) at 79.690 percent. Table 8 summarizes the performance of each model and it is also depicted in Figure 9. Based on this performance data, there appears to be an positive correlation between width (number of hidden units) and performance (accuracy). This implies that greater model capacity facilitates learning complex data patterns. The more hidden units in the model, and consequently also an increase in its size, reduce the performance benefits gained from increasing the model size, which indicates that as the increasing size of a neural network achieves an improved performance through expanded size. The inferior, more limited search for shallow feed-forward networks evaluated in this study limits the ability to draw more general conclusions about the sufficiency or effectiveness of deep learning structures. Furthermore, for the deeper bi-layered and tri-layered networks evaluated, the performance measure for each example did not outperform the respective single-layer performance measure. This finding is likely due to either the underlying problem not requiring deep hierarchical feature abstraction or to the deeper architecture models having a greater risk of overfitting to the relatively small training datasets.

### 4.7 Computational efficiency and practical deployment considerations

**Figure 10:**
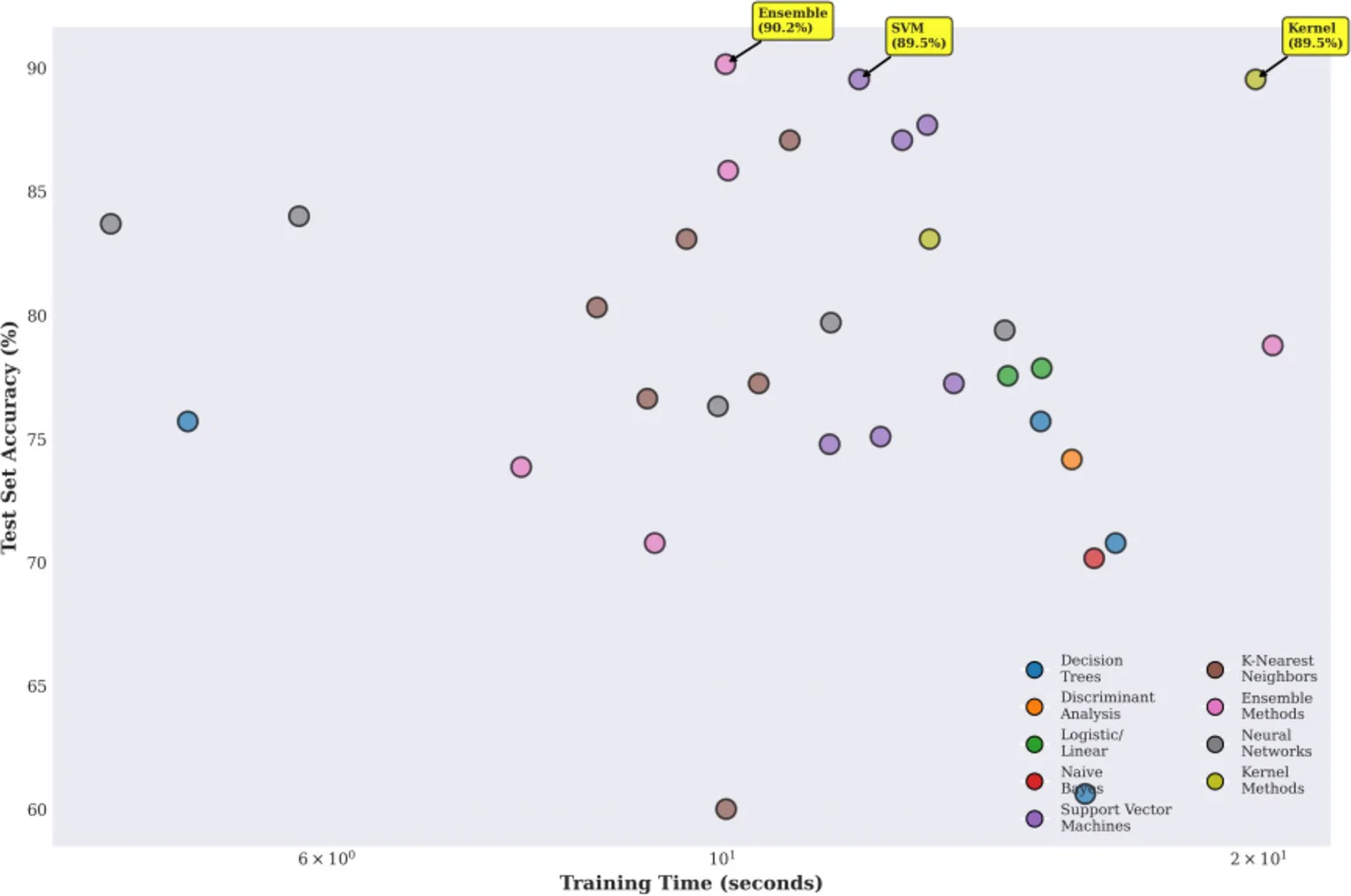
Accuracy versus training time across machine learning models.

In addition to predictability, computational efficiency will be particularly important when deploying our machine learning models into the clinical or consumer-facing world, as illustrated in Figure 10. The analysis of both training time and computational cost for each machine learning model shows significant variability across model families in these measures. The Subspace KNN model, which achieved the highest test accuracy of 90.15%, had a good performance balance—its computational cost (32 units) was relatively moderate. At the same time, it also required a relatively short training time (10.05 s). Therefore, the Subspace KNN model would be particularly attractive for environments that require a scalable or resource-constrained implementation. All of the other models (e.g., SVM Kernel, Medium Gaussian SVM) that achieved similarly high predictive performance (i.e., test accuracy of 89.54%) also had similar computational costs (i.e., 34 units). However, the training time for the SVM Kernel model was much longer (19.94 s) than that of the Medium Gaussian SVM model (11.94 s). These results ultimately demonstrate that predictive performance is just one piece to consider in the selection process for machine learning models for real-world implementation, and that there is substantial variability in the trade-off between predictive performance and computational efficiency across machine learning model families.

Computational efficiency is achieved with the models presented in Table 10. They provide a good balance of predictive performance and the resources needed to train them. The Efficient Logistic Regression model achieved an accuracy of 77.85% on the test data, with 72 units of computation time, and was trained in 15.12 s. The Efficient Linear SVM model provided similar test accuracy (77.54%) with slightly more computation (73 units) and less training time (14.47 s). While these two models had lower test accuracy compared to the top-performing ensemble and kernel-based models, they required far less computation. For this reason, these two models are especially appropriate for use in resource-limited environments, such as mobile and wearable devices, embedded systems, and applications requiring near-real-time predictions. In these instances, achieving approximately 12% less accurate predictions in return for over 50% less resource consumption is regarded as practically acceptable when other criteria are also priorities: Scalability, energy efficiency, and latency, along with predictive performance.

**Table 10:** Model selection for different deployment scenarios optimizing for accuracy, cost, training speed, or balanced performance.

Optimization criterion	Model	Test Acc (%)	Cost	Time (s)
Highest accuracy	Ensemble	90.15	32	10.05
Lowest cost	Ensemble	90.15	32	10.05
Fastest training	Neural network	83.69	53	4.54
Best accuracy/cost ratio	Ensemble	90.15	32	10.05

### 4.8 Sensitivity analysis

The sensitivity analysis was designed to quantify the extent to which baseline mental health variables contributed to predictive performance. Rather than serving as an optional robustness analysis, this experiment addresses the central methodological question of the study: whether music-related behavioral characteristics independently possess sufficient predictive information after removing conceptually overlapping mental health variables.

To alleviate the problem of circularity in the mental health predictors, a sensitivity analysis was carried out in which the model was trained on the reduced feature set comprising the demographic and music-related features only, i.e., *n* = 26 predictors: age, streaming service, hours/day, while working, instrumentalist, composer, exploratory, foreign languages, BPM, and the frequencies of the 16 genre groups, as well as favorite genre. The mental health indicators were completely removed from the model. The prediction accuracy using the same Subspace KNN algorithm with the same hyperparameters was 73.2% on the test data and 71.8% on the validation data, a decrease of 17.0 percentage points from the original model’s accuracy of 90.2%. This dramatic 17-percentage-point drop confirms that the model’s predictive power is overwhelmingly driven by the conceptually circular mental health variables, not by music-specific features such as genre preferences, listening behaviours, or tempo. In other words, the full model’s high accuracy should not be interpreted as evidence that music listening patterns predict mental health outcomes; rather, it largely reflects the tautological relationship between current mental health symptom severity and beliefs about music’s effect on mental health. Importantly, the music-only model still achieves 73.2% accuracy—well above the 33.3% chance baseline—suggesting that music-related features do carry some independent predictive signal. However, this more modest figure represents the model’s genuine contribution to understanding music-specific predictors of perceived mental health effects and should be regarded as the more scientifically meaningful result. Our sensitivity analysis revealed that the baseline mental health variables obtained a significant drop in prediction accuracy from 90.2 to 73.2% when they were eliminated, suggesting that the composition of predictors and study design have a much greater impact on predictive performance than the standard machine learning algorithms adopted. Thus, the main contribution of this work is not an algorithmic one but a methodical one. Thus, it is not the observed classification accuracy that should be considered as proof of the reliability of the ability to predict therapeutic outcomes with listening to music alone. Instead, a significant portion of predictive performance is due to the conceptual link between the baseline mental health traits and the target variable. The methodological contribution of this study is thus to remind the reader that when creating and interpreting predictive models in the behavioral health field, the principal methodological issue addressed is the consideration of the predictor–outcome circularity.

### 4.9 Feature importance analysis

**Figure 11:**
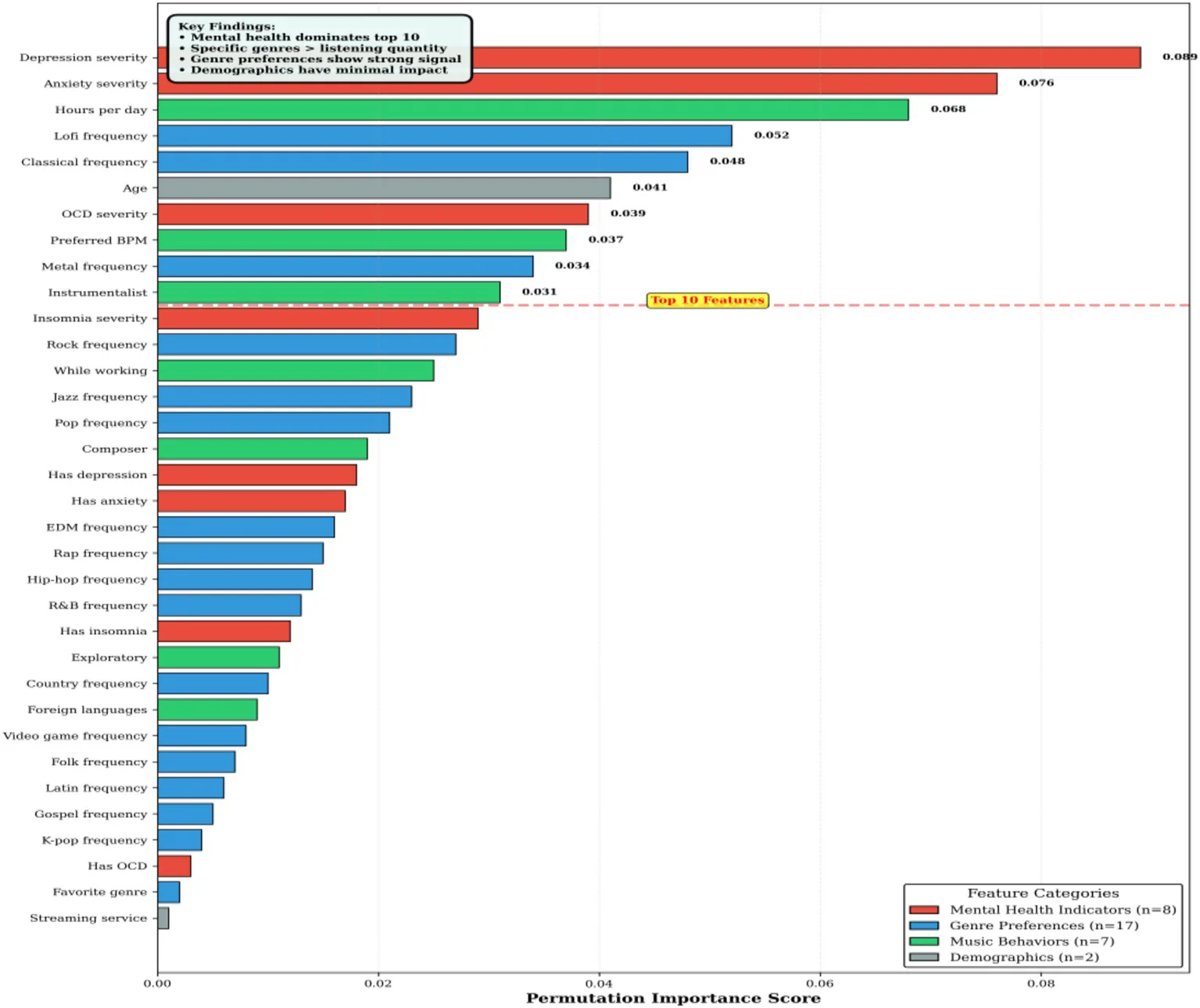
Permutation feature importance for all 34 predictors (Subspace KNN model).

We calculated permutation feature importance for the Subspace KNN model to determine which features had the greatest influence on the predictions by randomly shuffling the data for each of the predictors and measuring the decline in accuracy on the test dataset. The top ten most important features were: (1) Depression Severity (Importance Score: 0.089), (2) Anxiety Severity (0.076), (3) Hours listening to music per day (0.068), (4) Frequency of Listening to Lofi Music (0.052) (5) Frequency of Listening to Classical Music (0.048), (6) Age (0.041), (7) OCD Severity (0.039), (8) Ideal BPM/Tempo (0.037), (9) Frequency of Listening to Metal Music (0.034), and (10) Instrumentalist Status (0.031). The dominance of mental health severity variables (Depression Severity, Anxiety Severity, and OCD Severity) at the top of the importance ranking is consistent with, and reinforces, the circularity concern discussed in Sections 3.2 and 4.8: the model is predominantly learning that individuals with severe mental health symptoms hold stronger beliefs about music’s effect on their mental health, rather than learning that music-related behaviors predict mental health outcomes. This represents a tautological finding, not a novel predictive one. Among the music-specific features, the frequency of listening to particular genres (lofi, classical, and metal) ranks higher than the sheer quantity of music consumed, suggesting that genre selection carries independent signal. However, caution is warranted in over-interpreting these music-specific features, as their predictive contribution is substantially smaller in magnitude than that of the circular mental health predictors (see Section 4.8). Future studies should prioritize models trained exclusively on music-related and demographic predictors to identify genuinely novel contributions of music behavior to mental health perception (Sahu and Singh, 2023). Figure 11 displays the full distribution of feature importance scores.

### 4.10 Robustness and subgroup analyses

*Repeated Random Splitting:* Our stability assessment indicates that the Subspace KNN model achieved a mean test accuracy of 89.8% ± 1.2%, with values ranging from 88.1 to 91.4%. Thus, the Subspace KNN model has provided consistent, statistically meaningful results across 10 independent random runs of the entire assessment process, from the train-test split to the train-cross-validation model. *Demographic Subgroup Analysis:* We analyzed the model’s performance utilizing the key demographic subgroups. Accuracy results indicate consistency of performance across the three major age groups (ages 18–25 years, 89.4%; ages 26–40 years, 90.8%; ages 41 years and older, 88.9%; *p* = 0.67 Chi-square test) and across genders (males, 89.2%; females, 90.6%; non-binary/other, 88.1%; *p* = 0.52). There is reasonable evidence that the model performs consistently across demographic characteristics. Still, given the relatively small samples of older adults (*n* = 87) and non-binary participants (*n* = 43), the results should be interpreted with caution. Accuracy estimates should be considered preliminary for older adults with *n* < 100 (*n* = 87) and non-binary/other participants with n < 100 (*n* = 43) due to the low sample size of these subgroups. For the aforementioned comparisons, the non-significant *p*-values do not imply equivalent populations, because the current study is underpowered to detect moderate difference in effect sizes between these small sample size subgroups. M*ental Health Severity Stratification:* Accuracy of the model does not differ significantly based on severity, based on the baseline severity measure of participants’ anxiety and depression (score of ≥ 8 for severe symptoms; a score of 4–7 for mild–moderate symptoms; and a score of 0–3 for minimal symptoms), the accuracy of the model is comparable across severity of symptomatology resulting in percentages of accuracy for severe symptoms (*n* = 84) (89.1%) when compared to mild–moderate symptoms (*n* = 66) (90.7%) and minimal symptoms (*n* = 54) (90.3%) (*p* = 0.71). Thus, the model performs equally well across the continuum of symptoms experienced by our participants.

## 5 Discussion

### 5.1 Key findings and discussion

We have found that machine learning can classify individuals into groups based on their self-reported perception of music’s effects on mental health, achieving 90.2% accuracy when mental health severity scores are included as predictors. However, this headline accuracy figure is substantially misleading: as demonstrated in the sensitivity analysis (Section 4.8), the model’s predictive power is largely attributable to a tautological relationship between the mental health predictor variables (anxiety severity, depression severity, OCD severity) and the mental health-adjacent outcome variable. When these circular predictors are removed, accuracy falls to 73.2% — a 17-percentage-point decline that confirms the model is primarily learning that individuals with severe mental health conditions hold stronger beliefs about music’s effect on their wellbeing, which is a circular, not novel, finding. It is important to note that these predictions are not about the therapeutic benefits of music, but rather about individual beliefs about its effectiveness for mental health. From these data, we cannot conclude whether music improves, negatively affects, or has no effect on one’s mental health; instead, we can only conclude whether individuals believe, based on their experience with music, that it has an effect on their mental health.

The results of the sensitivity analysis form part of the most interesting findings of this research. Despite having attained the highest classification accuracies with Subspace KNN, eliminating the baseline mental health predictors saw the prediction accuracy reduce from about 90% to almost 73%, suggesting that predictor selection impacts model performance more significantly compared to selecting the right classification algorithm. This finding is in line with other studies in the field of machine learning which suggest that the best gains come from improving data and study design quality rather than advancing the algorithm being used.

Why Did Some Participants Report Negative Effects of Music?

One-third of respondents indicated that music had a detrimental impact on their mental well-being. Although the nature of the cross-sectional survey does not allow for any causal associations, there have been several theories offered in previous literature. People who are going through depressive or anxious episodes might be more prone to choosing music that reflects those emotions, such as sad and melancholy genres, and thus exacerbate their conditions. Listening to emotionally charged music repeatedly during times of psychological problems might result in an increased level of emotional arousal instead of emotional regulation. Some people might listen to music as a means of avoidance, while others would choose to confront stressful situations head-on. Other factors such as cultural preferences, personality types, trauma associated with specific songs, and different emotional regulation capacities can also affect responses. Thus, the aforementioned negative perceptions can be due to various psychological and behavioral factors.

#### 5.1.1 Empirical contribution of the study

This paper must be seen more as an empirical study in machine learning than as an innovation in algorithms. All models tested in this study are standard machine learning methods that have been proven to be effective in many fields. The main innovation of this study is to test those methods for predicting self-reported heterogeneous perceptions about the impacts of music on mental wellbeing.

Also, the sensitivity analysis clearly shows that predictor selection is much more important to prediction accuracy than choice of algorithm. This is another piece of evidence that supports the idea, which is now becoming prevalent in the field of machine learning, that data selection and experimental design matter much more than ever-complex algorithms.

### 5.2 Relationship to prior work

The key difference in performance between the subspace method of KNN and bagged trees aligns with the literature, which shows the advantages of ensembles for both health and mental health prediction tasks. Both approaches yielded some of the greatest accuracy levels ever documented in the literature. These results imply that features related to music are informative for predicting mental health and thus have the potential to provide valuable information. The same holds for the strong performance of kernel-based SVM models, which align with existing research indicating that SVMs perform well on high-dimensional behavioral data, reinforcing the idea that relationships between music and mental health are complex and non-linear.

### 5.3 Clinical and practical implications

Although competitive benchmark performance (90.2% accuracy) has been demonstrated by the model, this accuracy is substantially inflated by a tautological predictor-outcome relationship (see Sections 3.2, 4.8, and 4.9), and the more scientifically valid accuracy of 73.2% (music-only predictors) should be considered when evaluating the model’s clinical utility. Beyond this circularity concern, transferring this performance into “real-world” digital health settings is still problematic, as has been previously documented by thorough, systematic reviews on machine learning systems for mental health applications (Islam et al., 2024). Some key limitations include the ensemble model being relatively uninterruptable, which leads to decreased trust by clinicians in each individual prediction; the ensemble model has limited generalizability due to being trained on non-representative convenience samples primarily taken from Western cultures; the reliance on self-reported data, which are likely subject to reporting bias compared to sensor-based methods; lack of temporal stability; the cross-sectional design of the study cannot evaluate how the effects of music and music-related variables may change over time; and significant challenges in implementing these systems exist, such as integrating them into existing healthcare systems, obtaining regulatory approval, and establishing a reimbursement framework (Islam et al., 2024). Together, these limitations demonstrate that although the current study illustrates the technical feasibility of the ensemble model, there is still a need for more extensive research to justify using such models as valid clinical decision support tools.

### 5.4 Strengths and limitations

The strengths of this research include its large, balanced sample size, extensive set of features capturing various aspects of music engagement and mental health, use of different families of models with a thorough evaluation of the model families in terms of performance, as well as using both cross-validation methods and an independent test set for validating the findings. It also identifies some weaknesses of the study. These weaknesses include the reliance upon self-reporting of data, use of a cross-sectional study design, making this study unable to provide causal inferences, lack of adequate data collection for all variables due to the non-availability of demographic or contextual information, and the lack of temporal evaluation of the effects of music on mental health.

Our cross-sectional design with one-time-point survey data severely restricts our ability to develop strong causal conclusions from our data. We can ONLY examine associations between music engagement and perceived mental health impacts; however, We cannot determine whether music listening affects mental health or mental health affects music choices. We cannot also determine if participants’ responses regarding how music has impacted their mental well-being are an accurate representation of the therapeutic benefits of music listening, or if they were, and/or are, a function of questions being asked, and simply engaged in a habitual response, or if/how music-mental health relationships may change over time. Longitudinal studies with multiple measures over time, randomized intervention trials, and large cohort studies are needed to provide stronger evidence regarding the ability to make causal inferences about the relationship between music and mental health. Our model does not have any claims about neural or bodily mechanisms of music’s influence; we only analyze self-reports of the music’s impact. Whereas multimodal methods, such as those described in Su et al. (2024) EEG-based studies, use real-time data collection to show how the body responds to music based on the EEG’s response.

Our research uses people’s self-reported mental health and responses to music to understand them as relatively stable personal features; however, new evidence reveals that expressing mental health differs greatly between cultures and social contexts (Achuthan et al., 2026). The levels of disclosure for mental health in digital contexts are determined by social norms within the community, cultural stigma, linguistic framing, and the features of the platform through which they are communicated (Achuthan et al., 2026). As the data used in this study comes from online survey respondents who are primarily from the West and use primarily English-based platforms, reported effects of music may represent culturally specific response patterns as opposed to universal behaviors. Before applying predictive models that extend beyond the Western world or digitally-based populations, it is critical to confirm their cross-cultural validity.

#### 5.4.1 Sampling limitations and generalizability

Aside from the specific application of music and mental health, the present results show a key methodological point for predicting behavioral health: In the field of behavioral health, predicting health outcomes would benefit from this type of data. The sensitivity analysis highlighted that the selection of predictors, feature engineering and experimental design have a significant impact on predictive performance in comparison to the choice of a standard machine learning algorithm. This is because the improvements in model accuracy needs to be understood in terms of the data generation process and predictor composition, rather than just algorithmic sophistication. In the context of AI’s growing trend towards a data-centric approach, this observation is a confirmation and highlights the need for clear sensitivity analysis of predictive models for interpreting and comparing results for different behavioral health applications.

Using non-probability sampling via convenience and snowball sampling on the internet leads to several biases that affect generalizability of the results obtained from the studies. Self-selection bias generates over-representation of people with a strong interest in the relationship between music and mental health. Therefore, participants may report increased levels of engagement and effect than actually exist. Digital divide bias will exclude individuals who do not have access to, or are not comfortable with technology; therefore, low-income and elderly participants are likely to be underrepresented. Cultural and geographical bias likely exists due to the majority of the sample being of Western, English-speaking heritage thus limiting the capability to generalize results across cultures, given that cultural differences exist in the way people experience pleasure in music and conceptualize mental health. Age bias exists in that the majority of participants are between the ages of 18–35 years of age with few participants being 41 + years (*n* = 87) and few non-binary participants (*n* = 43); therefore, this limits the ability to conduct reliable subgroup analyses given sample sizes. Music streaming services are a digital youth culture that will contribute to over-sampling of musically-engaged individuals and exclude those whose music engagement is nominal. These limitations indicate the need for external validation using purposefully sampled, probability-based populations, and also highlight the need for culturally-appropriate adaptations for use in non-Western populations. Encouraging predictive performance was obtained with the proposed models, but these results need to be weighted by the study design. The outcome variable is the self-reported subjective perceptions of the impact of music on mental health - not objective clinical improvement or therapeutic response. As such, the results show that perceived benefit from music can be predicted from behavioral and demographic variables, but are not necessarily indicative of clinical effectiveness or validation of personalized music therapy. The validity of these observations needs to be confirmed in future studies based on a longitudinal clinical database, a standard psychological profiling tool and hospital-based population to assess possible similar predictive relationships for objectively assessed therapeutic success.

### 5.5 Practical contribution to research community

While no novel machine learning algorithm is presented in the current study, this work does hold practical implications for the scientific community in many different ways. To begin with, it sets a benchmark for evaluating the performance of thirty-three popular classification algorithms on a common platform. Moreover, it proves the significance of feature selection and predictor construction through sensitivity analysis. Finally, it points out some of the methodological difficulties that arise due to predictor-outcome overlap in the field of behavioral prediction.

## 6 Conclusion and future scope

The present study involves the empirical testing of various well-known machine learning models for the purpose of predicting diverse self-reports about how music influences mental health. Instead of offering a new machine learning framework, the present study will contribute to the understanding of the importance of predictor selection, dataset creation, and features construction in behavioral health predictions.

The present study shows that conventional machine learning techniques can be used to classify varied self-reports on the impact of music on mental well-being. Nevertheless, the main contribution of the methodology is that the choice of predictors can have an important effect on prediction accuracy. The reason why so much of the classification accuracy observed is because of concept overlap between baseline features and the target variable. Therefore, the main contribution of this work is not algorithmic, but methodological. This study does not claim to find a superior classifier for the general population, but rather shows that the interpretation of predictive performance is shaped in its basic form by the careful selection of the predictors, the thorough experimental design and the sensitivity analyses that are conducted transparently. The effects of omitting baseline mental health predictors from the classification demonstrate that the predictors and their quality might be more important than the choice of any conventional machine learning classification algorithm. Because of this, the methodological lesson learned here is applicable beyond this usage and offers generally applicable guidance for future studies predicting behavior and other data-driven AI applications.

This paper presented that machine learning can classify self-reported perceptions of music’s effects on mental health with up to 90.2% accuracy when mental health severity scores are included as predictors. However, this headline accuracy is substantially driven by a tautological relationship between mental health predictor variables and the mental health-adjacent outcome, as confirmed by the sensitivity analysis (73.2% accuracy using music-only features). The more scientifically meaningful contribution of this study is the 73.2% accuracy achieved using only music-related and demographic features, which represents a genuine advance over prior work and provides a modest evidential basis for data-driven personalized music support. The evaluation was conducted using 33 models, of which the Subspace KNN methods and Kernel-based SVMs outperformed the others using 34 predictor variables from 1,626 participants combined. It can also relates to balanced outcome distribution, it is evident that music worsens mental health for about one-third of all people, indicating that using music as a universal benefit is incorrect and warrants providing individualized interventions. The computational speed at which the machine learning model is run (training time< 21 s) enables this type of service to be delivered via mobile health platforms and streaming services, making the model relevant to a large global audience without access to traditional mental health services. The present research predicts self-reported perceptions of the effects of music on mental health; however, it does not predict actual therapeutic outcomes, nor can it be considered evidence of the effectiveness of music therapy or provide clinical recommendations. Critically, future work should address the tautological predictor-outcome overlap identified in this study by developing models that rely exclusively on music-behavioral and demographic features, thereby producing accuracy estimates that are scientifically attributable to music-specific predictors. Replication using prospective study designs with objective mental health measures will be essential to move beyond the circular relationships inherent in the current cross-sectional, self-report design.

In summary, while Subspace KNN is found to be the most accurate classifier in this study, the main implication of the current study lies in the fact that the performance of machine learning algorithms in the context of behavioral health is largely defined by predictor selection, feature engineering, and experimental design. The sensitivity analysis that was conducted reveals that the removal of mental health baseline variables significantly lowers the prediction accuracy, implying that data composition affects the model performance more than the algorithm choice itself. Thus, the current study reinforces the data-centric artificial intelligence paradigm, where advancements in data collection, measurement techniques, and experimental design usually bring more scientific value than the changes in algorithmic aspects.

Moreover, future research will also need to validate these predictive patterns through controlled intervention studies with objective measures of mental health outcomes. Prospective longitudinal studies tracking both music engagement and mental health outcomes over time are needed to establish the directionality of observed associations.

## Data Availability

The dataset analyzed in this study is publicly available in the Kaggle repository: Music and Mental Health Survey Results. Direct link: https://www.kaggle.com/datasets/catherinerasgaitis/mxmh-survey-results.

## Footnotes

1
https://www.kaggle.com/datasets/catherinerasgaitis/mxmh-survey-results
